# Conformational Change of Nucleosome Arrays prior to Phase Separation

**DOI:** 10.21203/rs.3.rs-2460504/v1

**Published:** 2023-01-19

**Authors:** Meng Zhang, César-Díaz Celis, Jianfang Liu, Carlos Bustamante, Gang Ren

**Affiliations:** 1 The Molecular Foundry, Lawrence Berkeley National Laboratory, Berkeley, USA; 2 Applied Science and Technology Graduate Group, University of California, Berkeley, USA; 3 California Institute for Quantitative Biosciences, University of California, Berkeley, USA; 4 Howard Hughes Medical Institute, University of California, Berkeley, USA; 5 Department of Chemistry, University of California, Berkeley, USA; 6 Department of Physics, University of California, Berkeley, USA; 7 Department of Molecular and Cell Biology, University of California, Berkeley, USA; 8 Molecular Biophysics and Integrative Bioimaging Division, Lawrence Berkeley National Laboratory, USA; 9 Kavli Energy Nanoscience Institute, University of California, Berkeley, USA

**Keywords:** Nucleosome, DNA, fluctuation and dynamics, ionic strength, single-molecule 3D imaging, individual-particle electron tomography, IPET, cryo-electron tomography, cryo-ET, linker histone H1

## Abstract

Chromatin phase transition serves as a regulatory mechanism for eukaryotic transcription. Understanding this process requires the characterization of the nucleosome array structure in response to external stimuli prior to phase separation. However, the intrinsic flexibility and heterogeneity hinders the arrays’ structure determination. Here we exploit advances in cryogenic electron tomography (cryo-ET) to determine the three-dimensional (3D) structure of each individual particle of mono-, di-, tri-, and tetranucleosome arrays. Statistical analysis reveals the ionic strength changes the angle between the DNA linker and nucleosome core particle (NCP), which regulate the overall morphology of nucleosome arrays. The finding that one-third of the arrays in the presence of H1 contain an NCP invaded by foreign DNA suggests an alternative function of H1 in constructing nucleosomal networks. The new insights into the nucleosome conformational changes prior to the intermolecular interaction stage extends our understanding of chromatin phase separation regulation.

## Introduction

Throughout the cell cycle, chromatin undergoes a structural transition between two major states, *i.e.* a loosely packed transcriptionally active state and a tightly packed transcriptionally silent state^1^. The switching between these two states is achieved by the reorganization of nucleosome arrays. The nucleosome arrays, known as "10-nm" chromatin fibers, consists of tandem repeats^2^ of nucleosome core particles (NCPs)^3^ separated by 10-60-bp DNA linkers, while each NCP is composed of 147-bp DNA wrapped ~1.7 turns around the histone octamer. Several models have been proposed to explain the conversion between transcriptionally active “10-nm” fiber^4^ and its inactive condensed form^5^. For instance, the “10-nm” fiber has been observed to condense into various forms of “30-nm” fibers, including one-start solenoid^6,7^, two-start helical ribbons^8,9^, untwisted two-start helix^10^, and a crossed-linker^11^. Further hierarchical folding and intertwining of the “30-nm” fibers have been historically considered key intermediates in the attainment of the higher-order chromatin structures^12-14^ and chromatids^15,16^. However, the failure to consistently detect the “30-nm” fiber *in vivo*^17-20^ has challenged this hierarchical model. In contrast, a reversible thermodynamic process termed liquid-liquid phase separation (LLPS) was proposed to explain chromatin compartmentalization and its condensation behavior. This theory has been supported by evidence from both *in vitro* and *in vivo* experiments^21,22^.

**Extended Data Fig. 1:**
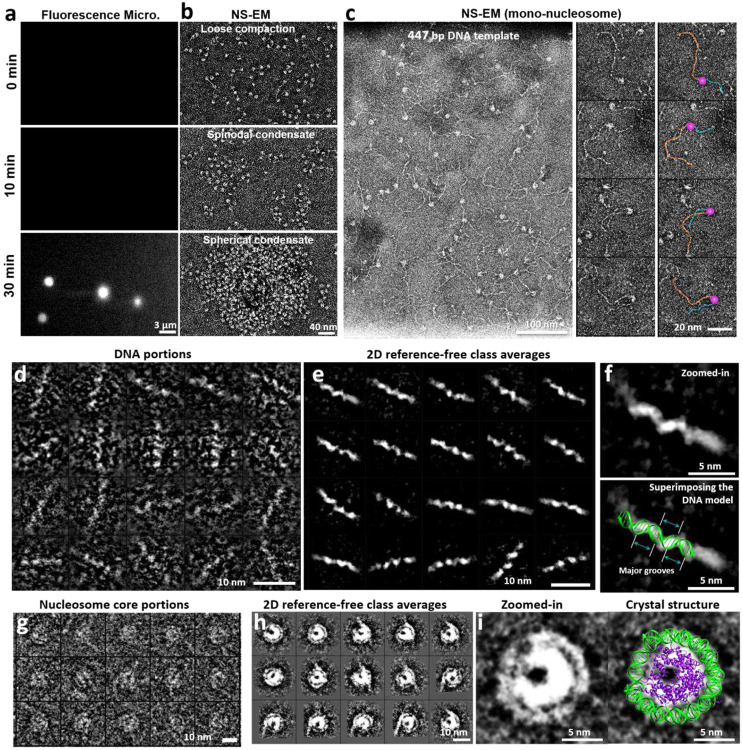
Morphology of mono-nucleosomes imaged by NS EM. **a,b** Development of nucleosomal condensates in physiological salt by fluorescence microscopy (a) and NS-EM (b) at 0-min, 10-min, and 30-min. **c**, Representative micrographs (left) and selected particles (right) of mono-nucleosomes reconstituted by assembling histone octamers on a 447-bp DNA template with 200-bp and 100-bp entry and exit DNA arms in low salt condition (5 mM Na^+^), respectively. In the schematic, the core particles, entry and exit DNA arms are marked by pink, orange and cyan, respectively. **d,e**, Representative images of DNA arms segments (**d**) and their reference-free class averages (**e**). **f**, Zoomed-in image of a representative averaged DNA segment (top) super-imposed with a standard DNA model (bottom), where its major groove is indicated by cyan arrows. **g,h**, Representative images of NCPs (**g**) and its reference-free class averages (**h**). **i**, Zoomed-in image of a representative averaged NCP particle superimposed with the crystal structure 1AOI.

**Fig. 1:**
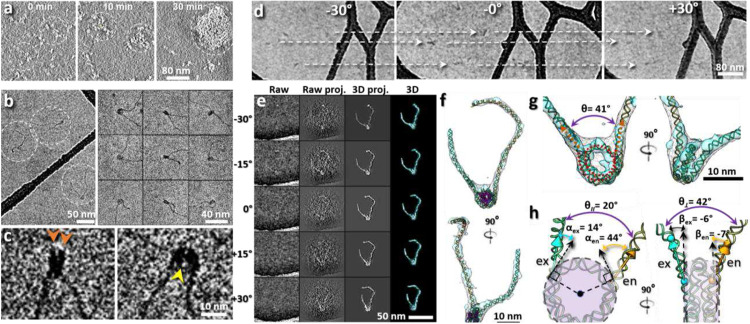
Cryo-EM images and 3D reconstruction of an individual mono-nucleosome particle. **a,** Representative cryo-ET time series of nucleosome arrays 3D reconstruction z-dimensional slice (14 nm) at the initial loosely packed (0-min), spinodal (10-min) and nucleation (30-min) states. **b,** Survey cryo-EM phase plate images (left) and selected particles (right) of reconstituted mono-nucleosomes flanked by 200-bp entry and 100-bp exit DNA arms in 20 mM HEPES buffer with 5 mM Na^+^. **c**, Zoomed-in phase plate images of two representative particles with detailed structural features: near two turns of wrapped DNA and the low-density center of the NCP are indicated by orange and yellow arrows, respectively. **d**, Representative cryo-ET tilt series, in which targeted particles are linked by dashed arrows. **e**, 3D reconstruction process of an individual nucleosome particle. The raw image, the projection of the mask-free initial 3D reconstruction, the projection of the masked 3D reconstruction, and the final 3D density map (column 1 through 4, respectively) of the target particle are compared at corresponding tilting angles. **f**, Perpendicular views of the final 3D map in (**e**) displayed at two contour levels and superimposed with its flexibly fitted model. **g**, Zoomed-in view of the NCP region of the fitted model in (**f**). ***θ*** corresponds to the angle between the two DNA arms. **h**, Schematic showing the projection of ***θ*** angle onto planes parallel (left) or perpendicular (right) to the NCP discoidal plane. The orientation of the linker DNA relative to the NCP is defined as the wrapping angle α and the bending angle β, for both entry (cyan) and exit (yellow) side of the NCP.

To understand the mechanism of the reversible LLPS process, tetranucleosome arrays (containing the 601-nucleosome positioning sequence, NPS) were frequently used as a standard sample for studying chromatin structures^3,23-26^ and their phase separation ^21,27,28^ in response to external stimuli, including ionic strength, temperature and protein factors, such as linker histone H1^19,21,27,29,30^. Recently, we have used the cryogenic electron tomography (cryo-ET) technique to study the early stage of tetranucleosome phase transition^28^ (Fig. 1a and Extended Data Fig. 1a-b). Through three-dimensional (3D) reconstruction of tetranucleosome condensates, we described a two-step phase transition mechanism ^28^ in nucleosomal LLPS. i) A spinodal decomposition process yields irregular condensates via intermolecular interactions among the nucleosome arrays. ii) Those condensates undergo unfavorable conversion into more compact, spherical nuclei, followed by growing into larger spherical aggregates through accretion of spinodal material or by fusing with other spherical condensates ^28^. In this process, Histone H1 catalyzes more than 10-fold the spinodal-to-spherical conversion^28^. This study implies that chromatin will naturally maintain or develop into a highly compacted state in physiological conditions unless hydrophobic histone modification or external stimulation are involved^21^.

Cells in their cycle spend 95% of its time in interphase^31^, where large portion of chromatins are loosely packed and accessible in the euchromatin region^32^. However, due to its intrinsic flexibility, a consensus structure model of the opening array is still missing. Thus it is necessary and of great interest to characterize the array structure at a loosely packed transcriptionally active state, and to visualize how the array conformation responds to external stimuli ^29^ prior to the intramolecular interaction and phase transition. Here, we extended the capability of cryo-ET to snap-shot the 3D structure of each individual particle of mono-, di- tri- and tetranucleosomes at a loosely packed transcriptionally active state, achieved under a low-ionic strength condition (5 mM Na^+^)^33^. Through quantitively measuring the conformations, we investigated the conformational change of nucleosome array dynamics affected by two independent factors, *i.e.* an increased salt concentration (up to 50 mM Na^+^) and the presence of linker histone H1. The changes observed could help us understand the reversible process of the LLPS mechanism for gene expression and regulation under external stimuli ^29^, especially for those prior to the reported intermolecular interactions and phase transitions.

## Results

### Flexible mononucleosome array particles

To obtain 3D snap-shot structures of individual nucleosome array particles requires the ability to directly image the NCP and the linker DNA without averaging. Due to its small physical diameter and flexibility, 3D reconstruction of individual DNA fragments is challenging^34-36^. Here, we employed both cryo-ET (Fig. 1b-c) and negative-stain ET (NS-ET) (Extended Data Fig. 1c) to test the 2D-imaging capability to determine the nucleosome positioning along a flexible DNA string. Histone octamers were assembled on a 447-bp-long DNA containing a 601-nucleosome positioning sequence (NPS). The 601-NPS facilitates the formation of highly positioned nucleosome arrays and thus has been used as a standard in a great number of nucleosomal structure studies^3,23-26^ and phase separation researches^21,27,28^. To determine the orientation of the nucleosome, we designed the entry DNA arms to be longer than the corresponding exit DNA arms.

Surveyed cryo-EM and NS-EM micrographs and their representative particles of mononucleosome in low ionic strength buffer (5 mM Na^+^) showed a “bead-on-string” structure with characteristic features, including i) a discoidal-shaped NCP with dimensions of ~10 × 10 × 4 nm (Fig. 1b-c) and DNA arms with a width of ~2 nm displaying a ~2 nm helical pitch corresponding to the major groove (Extended Data Fig. 1d-f; ii) nearly 2 turns of DNA wrapped around the histone disc (Fig. 1c, **orange arrow**); and iii) a low-density pore (~1 nm) present near the center of the nucleosomal disk (Fig. 1c yellow arrow and Extended Data Fig. 1g-i). These features are consistent with the NCP crystal structure (PDB entry 1AOI)^3^; likewise, the length of the DNA arms matched their expected values. This test demonstrates our nucleosome 2D-imaging capability, which became the basis for the following 3D reconstructions.

### 3D structure of an individual mononucleosome

**Extended Data Fig. 2:**
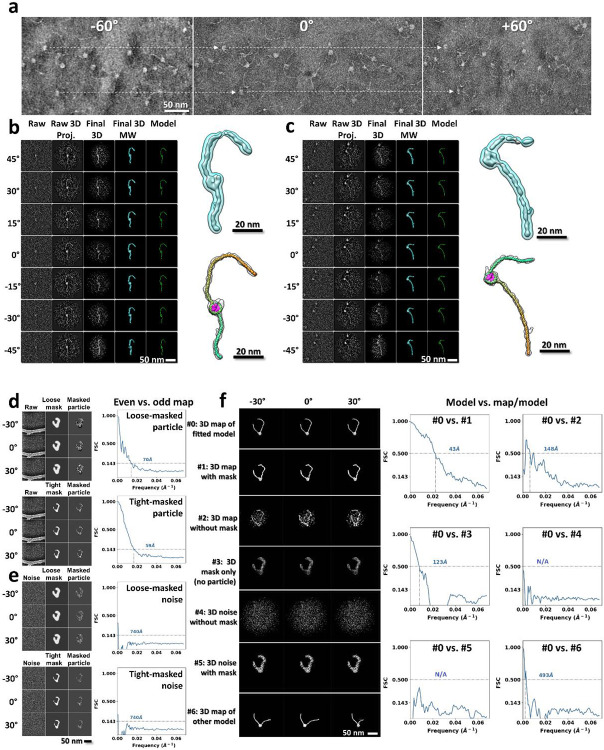
3D reconstruction of an individual mono-nucleosome particle and analyses of cryo-ET 3D reconstruction resolution. **a**, Representative tilt-series images of NS mononucleosome sample acquired by ET, in which targeted particles are linked by dashed arrows. **b,c**, 3D reconstruction of two mononucleosome particles. The raw images, the projections of the initial 3D reconstruction, the projections of the 3D reconstruction after IPET alignment, the final 3D density maps after missing wedge correction, and the flexible fitted models (column 1 through 5, respectively) of the target particles are compared at corresponding tilting angles. The zoomed-in image of the final 3D density maps and the same map superimposed with its flexible fitted model are shown on left panels. The histone, entry and exit DNA arms are colored in pink, orange and cyan, respectively. **d**, Resolution comparison showing that the reconstructions of cryo-ET samples are not sensitive to the size of mask used. A loose (top panel) and tight (bottom panel) particle-shaped mask are applied to the same aligned images before using their even and odd index of tilt series for 3D reconstructions and resolution estimation. **e**, Resolution comparison showing that masks alone do not contribute to the high resolution. The particle-shaped masks are applied to simulated noise images for 3D reconstructions and resolution estimation. **f**, Resolution evaluation by comparing the FSCs between the fitted model density (#0) with different maps (#1-6), including the final 3D reconstruction after masking (#1), the final 3D reconstruction before masking (#2), the 3D map of the mask (#3), the 3D reconstruction from the noise images (#4), the 3D reconstruction of the masked noise images (#5), and the 3D density map of another particle’s fitted model (#6). #1,3,5 used the same tight particle-shaped mask as in (**d**).

As a proof-of-concept to perform 3D-reconstruction of individual particles, we used mononucleosomes assembled on 447-bp DNA fragments (200-147-100). Reference-free 3D reconstruction by both cryo-ET (Fig. 1d-e) and NS-ET (Extended Data Fig. 2a-c) were performed using a method that combined individual-particle electron tomography (IPET)^37^, image denoising^38^, and missing-wedge correction by low-tilt tomographic reconstruction (LoTToR)^39^. The estimated resolution of up to 43 Å of the resulting 3D reconstruction was estimated based on different criteria and validated by additional analyses (Extended Data Fig. 2d-f). The reconstruction confirmed the “bead-on-string” conformation with structural details, such as the discoidal shape of the NCP (Fig. 1f), the two approximate turns of DNA wrapped around the histone core with defined entry and exit positions (Fig. 1g), and a measurable DNA arm length (Fig. 1f). These features allow us to determine the structure of each individual particle through rigid-body docking of the NCP crystal structure and the flexible fitting of the DNA arms, followed by connecting them together under energy minimization of molecular dynamics (MD) simulation^40^ (**Supplementary Video 1**). Since the structures obtained with NS-ET (Extended Data Fig. 2a-c) were relatively flattened due to the constraints imposed by the thin-layer stain, only the cryo-EM structures were used for further characterization.

The quantitative characterization of cryo-ET structures was conducted by measuring the spatial orientation of the DNA relative to the NCP according to the following parameters: i) wrapping length, *i.e.*, the length of DNA wrapped around the histone core (Fig. 1g, **red-dashed line**); ii) arm/linker angle *θ, i.e.* the angle formed between two vectors each aligned with 20-bp DNA segments of the entry and exit DNA arms extending from the NCP surface (Fig. 1g, **orange arrows**); iii) the two orthogonal projections of those vectors through angles, *θ*_∥_ and *θ*_⊥_, with respect to the discoidal plane of the NCP (Fig. 1h); iv) the wrapping angle *α* of the DNA arm vectors’ front-disc projections relative to the corresponding tangents of the discoidal plane (Fig. 1h, **left**); and v) the bending angle *β* of the vector’s side-disc projections relative to the disc side plane (Fig. 1h **right**). These parameters provided a basis for the statistical analysis of the nucleosome dynamics.

### 3D structural dynamics of mononucleosomes

**Fig. 2:**
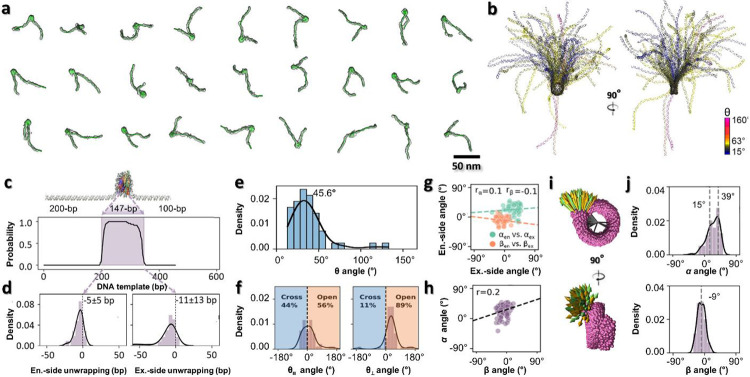
3D structure and dynamics of mono-nucleosome particles. **a**, 30 representative density maps reconstructed by cryo-ET from targeted mono-nucleosome particles in 20 mM HEPES buffer with 5 mM Na^+^, shown with fitted models. **b**, 47 super-imposed models after alignment based on their NCP portion, shown in orthogonal views. Models color-encoded by the ***θ*** angle between their two DNA arms. **c**, Histone octamer positioning along the 447 bp DNA template, which is presented as the probability of finding specific DNA base pairs in contact with the octamer surface (black line), calculated from 47 models. The 601-region is highlighted by a filled magenta box. **d**, Histograms showing DNA unwrapping distributions for the entry (left panel) and exit (right panel) side of the NCP. **e,f,** Histograms of the ***θ*** angle (**e**) and its two planar projections ***θ***_∥_ and ***θ***_*⊥*_ (**f**). **g**, Scatter plot showing a weak correlation between the entry and exit DNA arms for the same NCP, as indicated by the small r-value between *α*_*en*_ and *α_ex_* and between β_*en*_ and β_*ex*_. **h**, Scatter plot showing a weak correlation between the wrapping angle α and bending angle β measured from the same side of the NCP. **i**, Super-imposed vectors of the entry (yellow) and exit (green) DNA linkers on a consensus nucleosome model (magenta), shown in orthogonal views. **j**, Histogram of the wrapping angle *α* and the bending angle *β* distribution measured from all DNA linkers.

To delineate the dynamics of the mononucleosome 3D structure, a total of 47 density maps were reconstructed by cryo-ET (Fig. 2a, Supplementary Fig. 1 and Video 1), followed by modeling and characterization protocols described above. The ability to fit the DNA turns within the discoidal portion of the maps (**Supplementary Fig. 2**) enables the alignment of these models, which reveals highly dynamic DNA arms (Fig. 2b). Statistical analysis showed that the DNA wrapped around the histone octamer varied in length (Fig. 2c), consistent with cryo-EM single-particle averaging analysis^26^ and reflecting the “breathing” property inherent to NCP^41^. Compared to the crystal structure (1AOI^3^), the entry and exit DNA arms were unwrapped on average 5 ± 5 bp (mean ± standard deviation) and 11 ± 13 bp, respectively (Fig. 2d), which agree well with MD simulations showing DNA unwrapping in increments of 5 or 10 bp^42^. The NCP exit-side DNA arm displayed a more dynamic behavior than the entry-side, consistent with single-molecule fluorescence resonance energy transfer (FRET) experiments^43^, a property of the 601 DNA sequence with asymmetric affinities, in which the exit-side DNA is less flexible than the entry-side, leading to the former’s decreased affinity to the histone octamer.

The angle *θ* of NCPs are distributed over a wide range from ~0°-140° (with a peak population at ~39°, and a mean ± std of 46° ± 27°), indicating that the two DNA arms were relatively free to orient themselves with respect to one another (Fig. 2e). Its two components *θ*_∥_ and *θ*_⊥_ showed their peak populations at ~9° (with a mean ± std of 10° ± 44°) and ~32° (32° ± 45°) respectively (Fig. 2f), indicating that the two DNA arms were relatively parallel to the NCP discoidal plane, and only modestly bent away toward the plane’s normal direction. The weak correlation between the entry and exit *α* angles (Fig. 2g, **green points**), and between the entry-exit *β* angles (Fig. 2g, **orange points**) suggests that the two DNA arms move independently of one another. Thus, we merge entry- and exit-side measurements into a single distribution. Moreover, a very weak correlation between the *α* and *β* angles from the same DNA arm (Fig. 2h) implied no preferential DNA arm “swing trajectory” on the NCP surface (Fig. 2i). The *α* angles displayed a left-skewed distribution in a range of ~± 50° with major and minor peaks at ~39° (39° ± 5°) and ~15° (14° ± 16°), respectively (Fig. 2j), suggesting two low-energy states of the DNA arms along the NCP plane. The *β* analysis showed a near normal distribution in a range of ~30° with a peak population at ~ −9° (−9° ± 11°) (Fig. 2j), suggesting somewhat restricted dynamics of the DNA arms along the direction normal to the NCP plane.

To display the dynamics of the mononucleosome in solution, the 47 structures were pair-wise aligned based on minimization of their root-mean-square deviations (RMSD), followed by ordering them according to a hierarchical classification^44^ (**Supplementary Fig. 3**). Through interpolation of the intermediates between each two consecutive ordered structures by target molecular dynamics (TMD) simulations^40^, mononucleosomes are morphed from the most populated state to the rarest state, displaying the thermally-induced dynamics of the particles in solution (**Supplementary Video 1**). These results demonstrate the capability of the use of individual particle cryo-ET methods to study nucleosome array dynamics in the following studies.

### Effect of NCP number on nucleosome array 3D structural dynamics

**Extended Data Fig. 3:**
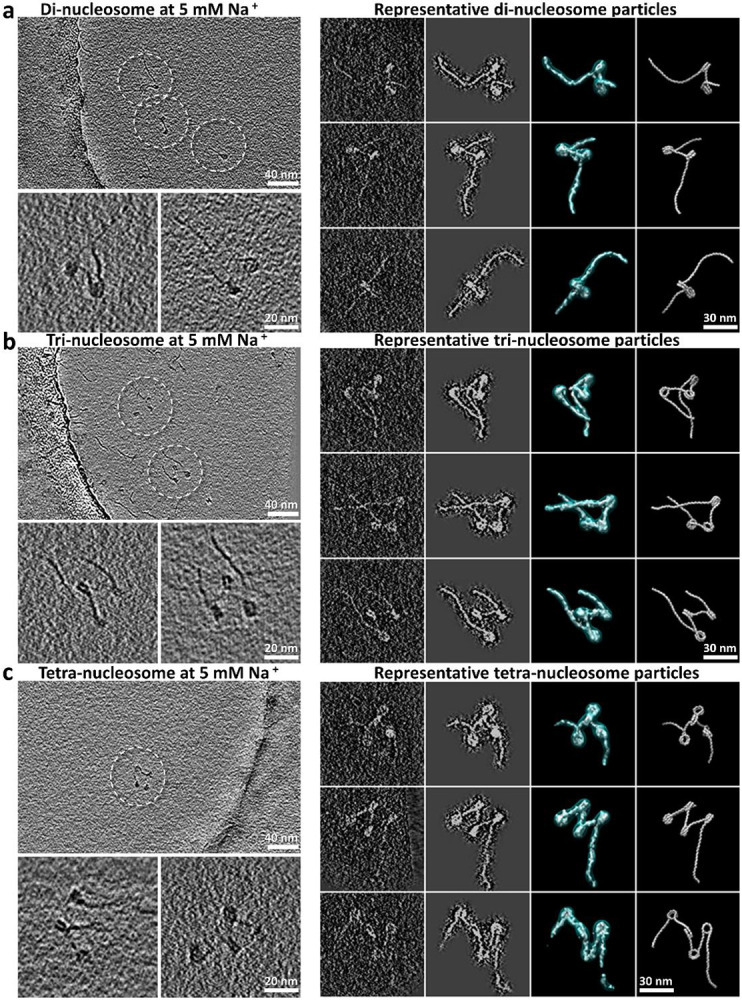
Cryo-ET 3D reconstructions of individual di, tri, tetra-nucleosome particles. **a**, Survey cryo-EM micrograph of reconstituted dinucleosome array in 20 mM HEPES buffer with 5 mM Na^+^ (top left panel). Target particles are highlighted by dashed-line circles. Two representative z-dimensional slices (3 nm thickness) of dinucleosome particle reconstructions are shown in bottom left panel. The 3D projection of the mask-free initial 3D reconstruction, the projection of the masked 3D reconstruction, the final 3D density map displayed at two contour levels, and the flexible fitted model of the targeted particle (column 1 through 4, respectively, right panel) are compared to each other. **b,c**, The same representation scheme is applied to the trinucleosome (**b**) and tetranucleosome (**c**) array samples in 20 mM HEPES buffer with 5 mM Na^+^.

**Fig. 3:**
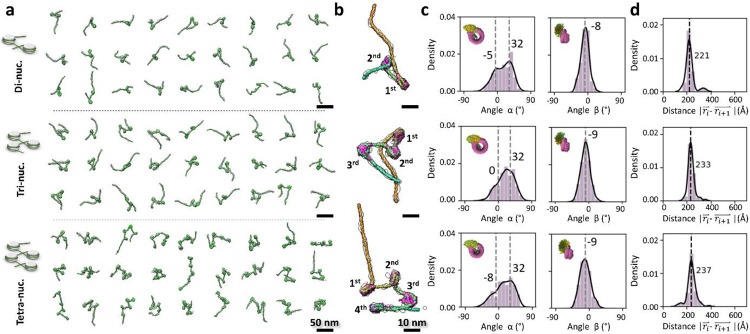
3D structures illustrating the dynamics of di-, tri- and tetra-nucleosome array particles. **a**, 27 representative cryo-ET density maps from individual particle reconstructions of di- (top), tri- (middle), tetra-nucleosomes (bottom) in 20 mM HEPES buffer with 5 mM Na^+^. Each of the density maps is super-imposed with its flexibly fitted model. **b**, Zoomed-in views of representative map and fitted model of each type of nucleosome array. DNA color-encoded by their bp index (from yellow to cyan) and histone colored in pink. **c**, Histograms of the wrapping angle α and the bending angles β. **d**, Histogram of the core-to-core distance measured between the *i* and *i*+1 NCP.

To investigate whether increasing the number of NCPs on a DNA segment changes the intrinsic structural dynamics of the resulting array, we repeated the above cryo-ET experiment using di-, tri- and Tetranucleosomes. These arrays were designed in a similar configuration as the mononucleosomes, *i.e.* 200-bp entry and 100-bp exit DNA arms. Additionally, a 40-bp DNA linker was inserted between two consecutive 147-bp 601 regions. We reconstructed a total of 33, 45 and 31 density maps of the di-, tri-, and Tetranucleosomes, respectively, followed by modelling and quantitative analysis as described above (Extended Data Fig. 3, and **Supplementary Fig. 4-8** and **Video 2**). These nucleosome array structures displayed an overall asymmetric zig-zag architecture (Fig. 3a-b) consistent with previously described nucleosome fiber conformations found within oligo-nucleosome samples^45^, cell nucleus sections^46,47^, and decondensed mitotic chromosomes^36^, but inconsistent with the symmetric structures previously proposed, such as the twisted double-helix revealed by cryo-EM single-particle averaging methods^24^ and crystallography^10,48^.

**Extended Data Fig. 4:**
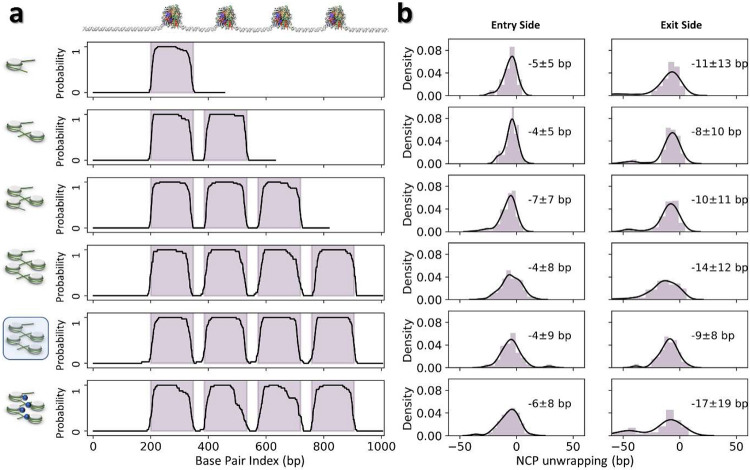
Analysis of nucleosome array unwrapping dynamics. **a**, Histone octamer positioning along the DNA template of mono, di, tri, tetranucleosome array at 5 mM Na^+^, tetranucleosome array at 50 mM Na^+^, and tetranucleosome array at 5 mM Na^+^ in the presence of H1 (row 1 through 6, respectively). The octamer positioning is presented as the probability of finding specific DNA base pairs in contact with the octamer surface (black line). The designed 601-regions on each DNA template are highlighted by filled magenta boxes. **b**, Histograms showing the corresponding DNA unwrapping distributions on the entry (left panel) and exit (right panel) side of all NCPs in (**a**).

The wrapped DNA at the entry-sides of the NCP units from di-, tri-, and tetranucleosome arrays were shorter than that of the crystal structure by −4 ± 5-bp, −7 ± 7-bp, and −4 ± 8-bp (mean ± std), respectively, and at the exit-sides shorter by −8 ± 10-bp, −10 ± 11-bp, and −14 ± 12-bp, respectively (Extended Data Fig. 4, **row 2-4**). The similar unwrapping levels compared to the mononucleosome (*i.e.* −5 ± 5-bp and −11 ± 13-bp for the entry and exit sides, respectively) indicate that the breathing motion in arrays is also controlled by the difference in asymmetric stiffness between the entry and exit sites^26,42,43^.

**Extended Data Fig. 5:**
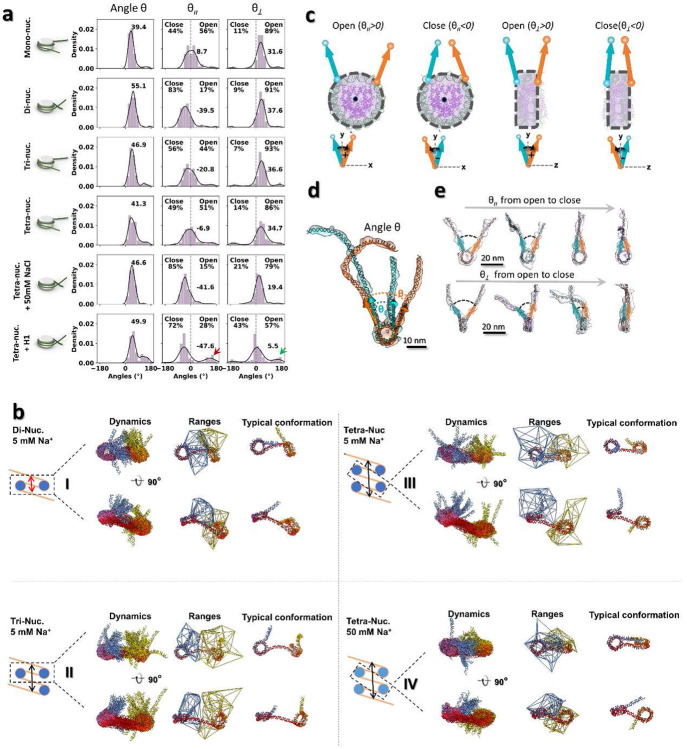
Distribution of the nucleosome array *θ* angles and analysis of array central dinucleosome unit morphology. **a**, Distribution of ***θ*** angle between two DNA linkers (first column) measured from all NCP units of different type of nucleosome arrays (schematic on left). The corresponding ***θ***_∥_ and ***θ***_*⊥*_ fraction are shown on second and third column, respectively. **b**, The central dinucleosome unit dynamics of di (I), tri (II), and tetra-nucleosome (III) array in 5 mM Na^+^ and the latter in 50 mM Na^+^ (IV). The central di-nucleosome components (40-147-40-147-40, dashed-line box) of arrays within the same category are extracted and aligned by minimizing their NCPs RMSD (left schematics). The red arrow marks the close distance between two distal DNA arms in dinucleosome array in contrast to those spatially separated arms presented in other type of arrays (black arrows). The DNA linker/arm conformational dynamics, dynamic range, and their typical conformation of each array categories are shown in column one through three. The DNA are rainbow colored from blue, red to yellow along the DNA entry-exist direction. **c**, Definition of the open-closed arm/linker conformation of an NCP by the sign of its ***θ***_∥_ and ***θ***_*⊥*_ fraction. **d,e**, Representative 3D reconstructions showing the dynamic of the ***θ*** angle (**d**) and its two fractions in orthogonal views (**e**) (planes parallel and perpendicular to the NCP discoidal plane, respectively).

Unexpectedly, we find that for nucleosome arrays, the number of NCPs is negatively correlated to the *θ* angle peak position, *i.e.* ~55°, ~47° and ~41° for di-, and tri- and tetranucleosomes respectively, and that its *θ*_∥_ component is more sensitive to the NCP number than the *θ*_⊥_ component (Extended Data Fig. 5a, **row 2-4**). The dispersion of *θ*_∥_ spans a range of ~33° (from −40° to −21° and −7° for di-, tri-, and tetranucleosomes, respectively), significantly larger than that of *θ*_⊥_, i.e. ~3° angle (from −38° to −37° and −35° for di-, tri-, and tetranucleosomes, respectively) (Extended Data Fig. 5a, **row 2-4**). Among these arrays, the dinucleosomes displayed the most deviated *θ* and *θ*_∥_ peaks compared to the mononucleosome, a feature that may result from the fact that it is the only array structure whose two negatively charged long distal (entry and exit) arms lie in close proximity and repel each other (Extended Data Fig. 5b, **panel I, red arrow**). In this case, the absence of a third and/or a fourth intermediate NCP separating the two repulsive entry and exit DNA arms induce a unique conformation of dinucleosome, displaying a noticeably restricted dynamic range and a twisting between two NCP discoidal planes (Extended Data Fig. 5b, **panel I**). By defining *θ*_∥_ < 0 as ‘closed-arm’ conformations (Extended Data Fig. 5c-e), ~83% of DNA linkers were found to adopt the closed forms (Extended Data Fig. 5a). These unique dynamics of dinucleosomes may relate to the ability of remodelers to recognize them. For example, it has been reported that Isw1a prefers to bind dinucleosomes rather than mononucleosomes and its binding affinity to the dinucleosome can be elevated by increasing the distal DNA arm length of the array regardless of histone modifications^49^. Also, SWI/SNF shifts the dinucleosome positions faster than those of trinucleosomes, as shown by electrophoresis assays^50^.

**Extended Data Fig. 6:**
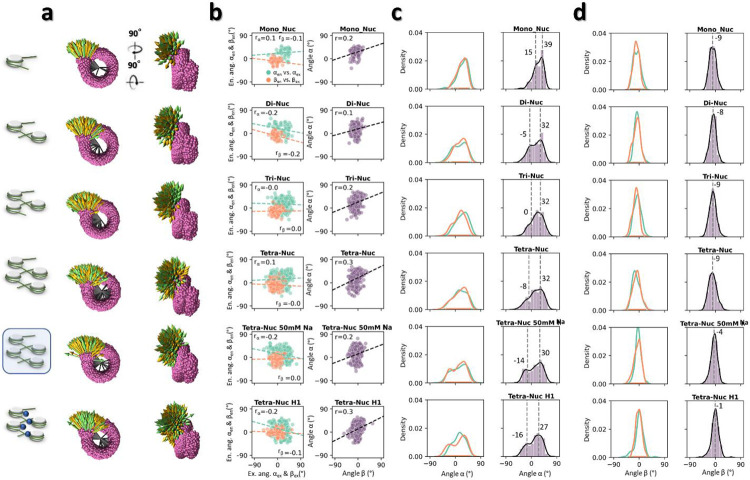
Distributions of the wrapping angle α and the bending angle β. **a**, Super-imposed vectors of the entry (yellow) and exit (green) DNA linkers on a consensus nucleosome model (magenta) for each array categories (left schematics), shown in orthogonal views. **b**, The Pearson correlation analysis between the two wrapping angles, α_en_ vs. α_ex_ (cyan) and between the two bending angles, β_en_ vs. β_ex_ (orange) on the same NCPs within the corresponding array category (left column). The correlation analysis is also performed between the same side of wrapping angle and bending angle of NCPs (purple) within each category (right column). **c**, Distribution of α_en_ and α_ex_ shown in cyan and orange, respectively (left column) and their pooling (right column). **d**, Distribution of β_en_ and β_ex_ shown in cyan and orange, respectively (left column) and their pooling (right column).

Although the *β* angle displayed no observable change with NCP number (all peaks were at −9°- −8°), the *α* angles of nucleosome arrays showed a negatively skewed distribution with two peaks: a major peak at ~32°, similar to the value for mononucleosomes (39°), and a minor peak between −8° and 0° not observed for mononucleosomes (15°) (Fig. 3c and Extended Data Fig. 6, **row 1-4**). A large positive *α* angle corresponds to the DNA conformation that bends away from the NCP discoidal plane, while a small negative *α* angle suggest that DNA arms could also slightly curve inward after lifting off from the NCP discoidal plane. The shift of the minor peaks from positive to negative values with increasing NCP number likely indicates that the DNA bending at entry and exit positions of the NCP is slightly reduced by the presence of nearby NCPs^51,52^.

**Extended Data Fig. 7:**
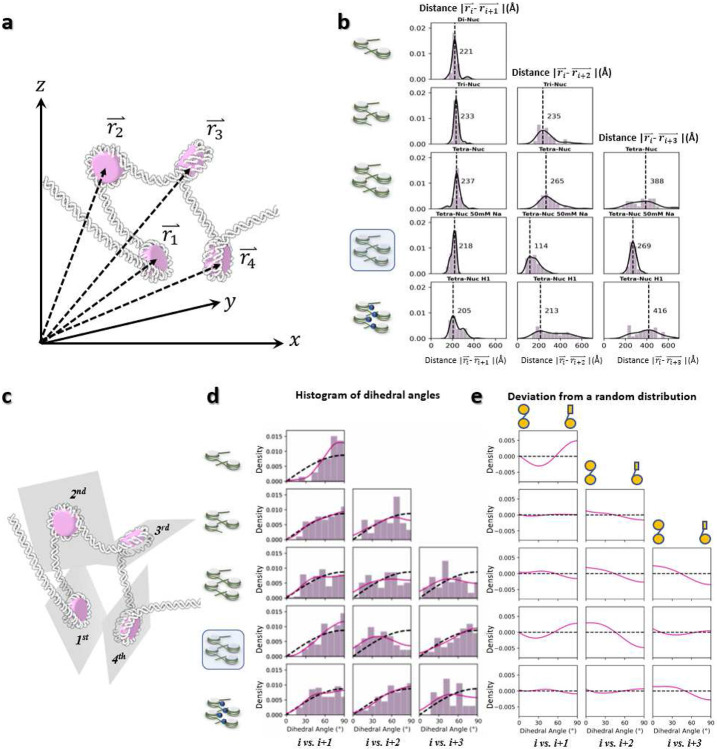
Distribution of the intra-nucleosome array NCP distances and dihedral angles. **a**, Schematics of the vector, $\vec{r_{ı}}$ starting from the origin and pointing to the center of mass of each NCP. **b**, Histograms of the measured distances between each pair of NCP centers within the array. **c**, Schematics defining the NCP central discoidal planes within the array. **d**, Histograms of the dihedral angle between each pair of NCP discoidal planes. The measured angle distributions (pink line) are compared with a sin function (black dashed-line, presenting the angle distribution of a randomly rotated plane against a fixed plane). **e**, The deviation of the dihedral angle distribution from the sin function indicates a preferred core-to-core conformational arrangement (schematics on top of each column).

Further analysis of the distance between consecutive NCPs (*i vs. i+1*) among arrays at different lengths showed that NCP spacing is independent of the NCP number and mainly controlled by the distance between the NPS. (Fig. 3d and Extended Data Fig. 7b, **row 1-3**). The analysis of the dihedral angle between consecutive NCPs showed no preferred orientation between their discoidal planes except for dinucleosomes, in which the two NCPs preferred a perpendicular arrangement (Extended Data Fig. 7d-e, **row 1-3**). The lack of a preferred orientation of the former, despite the high torsional rigidity of the DNA (torsional persistence length of ~90 bp), most likely reflect the dispersion of the *α* and *β* angles observed in our analysis. The defined orientation observed for dinucleosomes, on the other hand, may reflect the narrower dispersion in the *α* and *β* angles for this species, probably as a result of the interaction of the two distal DNA arms. This perpendicular conformation is consistent with the crystal structure of pseudo dinucleosome bound to Isw1a^53^, which suggest, in turn, that the factor recognizes or stabilizes it upon binding.

To display the dynamics of nucleosome arrays in solution, movies of di- and trinucleosomes were also created by the same protocol used for the mononucleosomes (**Supplementary Fig. 5 and 7** and **Video 2**). Above experiments suggest the increased number of NCP on a DNA segment did not dramatically change the intrinsic structural dynamics of the resulting array, except the NCP at two distal ends.

### Effect of ionic strength on the 3D structural dynamics of tetranucleosomes

**Extended Data Fig. 8:**
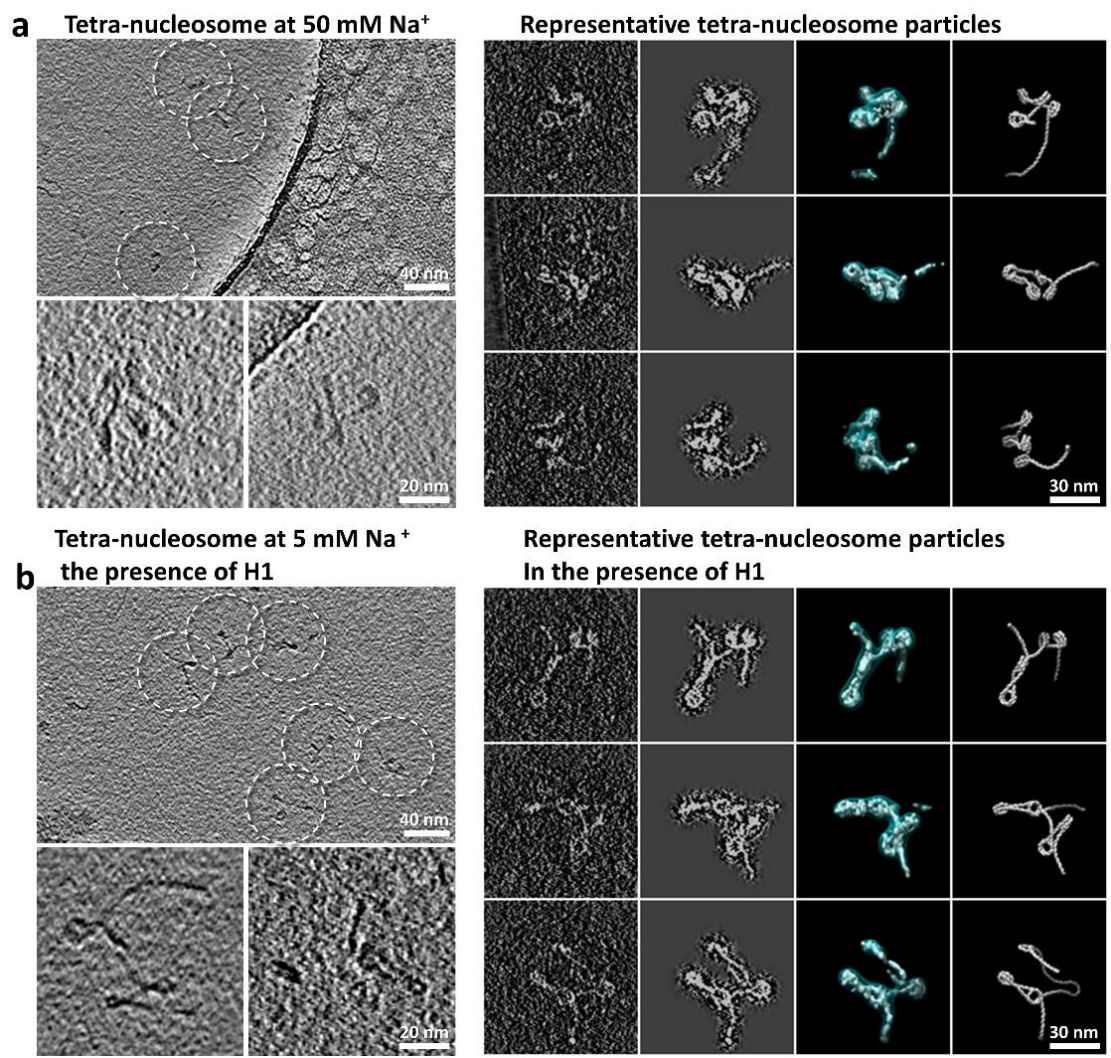
Cryo-ET 3D reconstructions of tetranucleosome particles in high-salt buffer and in the presence of H1. **a**, Survey cryo-EM micrograph of reconstituted tetranucleosome array in 20 mM HEPES buffer with 50 mM Na^+^ (top left panel). Target particles are highlighted by dashed-line circles. Two representative z-dimensional slices (3 nm thickness) of tetranucleosome particle reconstructions are shown in bottom left panel. The 3D projection of the mask-free initial 3D reconstruction, the projection of the masked 3D reconstruction, the final 3D density map displayed at two contour levels, and the flexible fitted model of the targeted particle (column 1 through 4, respectively, right panel) are compared to each other. **b**, The same representation scheme is applied to the tetranucleosome array samples in 20 mM HEPES buffer with 5 mM Na^+^ in the presence of H1.

**Fig. 4:**
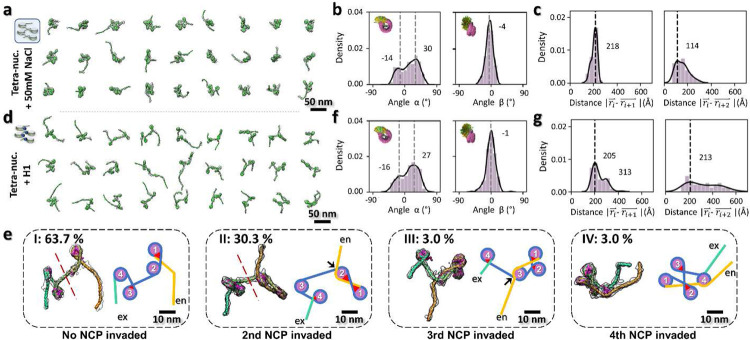
3D structures and dynamics of tetra-nucleosome arrays in 50 mM Na^+^ and in 5 mM Na^+^ in the presence of H1. **a**, 27 representative cryo-ET density maps reconstructed from individual tetra-nucleosomes in 20 mM HEPES buffer with 50 mM Na^+^. Each map is superimposed with a flexibly fitted model. **b**, Histogram of the wrapping angle α and the bending angle β distributions of tetra-nucleosome under high ionic strength. **c**, Histograms showing the distributions of the core-to-core distance between *i*, *i*+1 NCPs and *i*, *i*+2 NCPs. **d**, 27 representative density maps and models of tetra-nucleosome arrays in 20 mM HEPES buffer with 5 mM Na^+^ and in the presence of linker histone H1. **e**, Zoomed-in views of representative tetra-nucleosome array maps and models (left side of each panel) displaying four typical (I, II, III, and IV) NCP unwrapping/rewrapping conformations in response to the presence of H1. The schematics (right side of each panel) show various 200-bp DNA arm trajectories during its invasion to one of the intermediate NCPs. The entry-, intermediate-, and exit-DNA portion are colored in orange, blue, and green, respectively. Histones are colored in purple. Red dashed line indicates that the second NCP unwrapping yields two spatially separated di-nucleosomes, which are favor conformations (I and II). Red triangles mark the conventional H1 binding sides on NCP, while black arrows indicate the possible H1 binding sites introduced by the distal DNA arm invasion. **f,g**, Histograms of α and β angle distributions (**f**) and core-to-core distance distributions (**g**) measured from tetranucleosome NCPs in the presence of H1.

Tetranucleosome has been used as a phenotype with its minimal structural unit^54^ and its capability of forming higher-order chromatin structures including helical fibers^24,48^ and phase separated condensates^28^. In the previous study^29^ we showed, under physiological salt concentration (150 mM Na^+^ and 5 mM Mg^2+^), tetranucleosomes conduct a phase transition through intermolecular interaction. To investigate how tetranucleosomes initialize the intermolecular interaction prior to the early stage of phase transition ^29^, we repeated the cryo-ET experiment described above by slightly increasing the salt concentration to 50 mM Na^+^ (Extended Data Fig. 8a), and achieved a total of 34 density maps with their fitted models (Fig. 4, **Supplementary Fig. 9 and Video 3**). These structures again confirmed the asymmetric zig-zag arrangement of the arrays but displayed a more compacted shape in contrast with the structures observed at 5 mM Na^+^.

The extent of DNA unwrapping at the entry-side remains invariant with ionic strength, *i.e.* −4 ± 9-bp *vs.* −4 ± 8-bp for 50 mM and 5 mM, respectively, but the interaction at the exit-side was slightly stabilized by the increase of Na^+^ (−9 ± 8-bp *vs*. −14 ± 12-bp, for 50 mM and 5 mM respectively) (Extended Data Fig. 4b, **row 4-5**). Although *θ* angle is not sensitive to the increase in ionic strength (changing its peak position from 41° to 47°), its two perpendicular components *θ*_∥_ and *θ*_⊥_ display a significant peak shift with the increase of Na^+^ (from −7° to −42° and 35° to 19°, respectively) yielding a high percentage of closed-arm conformations (Extended Data Fig. 5a, **row 4-5**). Although the major peak of *α* remained unchanged, its minor peak became more apparent and shifted from −8° to −14° (Fig. 4b and Extended Data Fig. 6c, **row 4-5**). This transition may arise from the stronger electrostatic screening effect^55^, which reduces the repulsive interactions between the two DNA linkers, allowing them to become closer together. Consistently, the DNA linkers bend toward the NCP discoidal plane (due to the change of *β* from −9° to −4°, Fig. 4b and Extended Data Fig. 6d, **row 4-5**). Although further analysis showed a slight reduction in the distance between consecutive NCPs (*i vs. i+1*), (from 237 Å to 218 Å) with increased ionic strength, a significant distance reduction was found between every-other NCPs (*i vs. i+2*, from 265 Å to 114 Å), and between the first and fourth NCPs (*i vs. i+3*, from 388 Å to 269 Å) (Fig. 4c and Extended Data Fig. 7b, **row 3-4**), indicating a compaction process of the overall array conformations (Fig. 4a). Meanwhile, the dihedral angle between NCPs showed that, although the consecutive NCPs adopted a more perpendicular orientation with respect to each other, every-other NCPs turned to more parallel packing in contrast to the observations at low salt concentrations (Extended Data Fig. 7e, **row 3-4**). This experiment suggests the ions increased the electrostatic effect by inducing two DNA linkers to get closer to each other, resulting in the NCP packing towards to each other, resulting in shrinking the overall diameter.

### Effect of H1 on the 3D structural dynamics of tetranucleosomes

**Extended Data Fig. 9:**
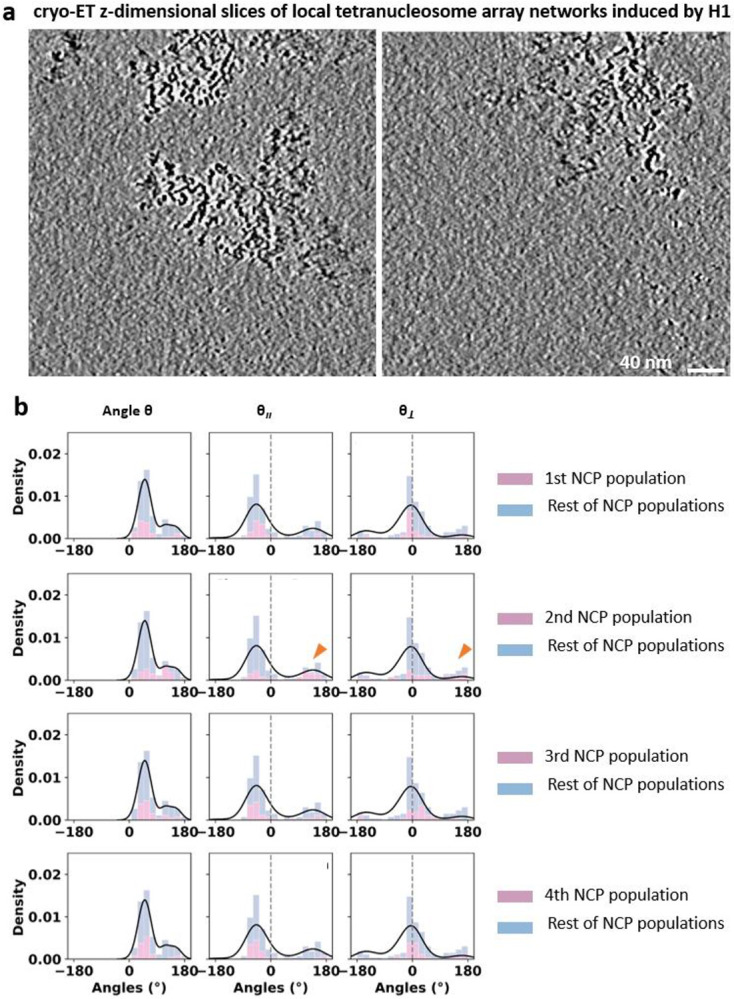
Linker histone H1 inducing inter-array local networks and causing intra-array NCP units’ differentiation. **a**, Cryo-ET z-dimensional slices (5nm thickness) of two representative area showing H1 can induce few tetranucleosome local networks in 20 mM HEPES buffer with 5 mM Na^+^. **b**, Distribution of NCP ***θ*** angles and its two perpendicular fractions measured from tetranucleosome array in the presence of H1 (column 1 through 3). The contribution of each NCP unit to the sub-population (minor peak of the distribution) is plotted individually in pink color (row 1 through 4). The orange arrows indicate that the minor peak is mainly caused by the second NCP.

Considering the Histone H1 catalyzes the spinodal-to-spherical conversion^28^ for more than 10-time ^29^ faster than that absents H1, it is interesting to investigate how H1 alone contributes to speeding-up the conversion during the regulation of the structural dynamics of the arrays. We incubated tetranucleosomes with H1 in a molar ratio of 1:4 under the same low-salt conditions (5 mM Na^+^). A total of 33 cryo-ET density maps with their models were obtained (Extended Data Fig. 8b, **Supplementary Fig. 10 and Video 3**). Once again, these arrays displayed a general asymmetric zig-zag conformation (Fig. 4d). However, these structures seem partially intertwined compared to arrays observed under low-salt without H1 (Fig. 3a), but less compacted than those imaged under high-salt without H1 (Fig. 4a). Interestingly, ~40% of the particles displayed a conformation in which the 200-bp DNA arm invades one of the NCPs by replacing part of its originally wrapped DNA at the exit side (Fig. 4e). This invasion can be observed within an array as well as between arrays, leading to the formation of larger structures (Extended Data Fig. 9a), reminiscent of the previously reported alternative function of H1 in strengthening the inter-array network^56^.

The analysis showed that the presence of H1 did not change the amount of DNA wrapping at the entry side (−6 ± 8-bp *vs*. −6 ± 8-bp) but slightly increased the unwrapping level and dynamics on the exit side (−17 ± 19-bp *vs*. −14 ± 12-bp) and generated a second peak at ~−45-bp (Extended Data Fig. 4a-b, **row 6**). This change in length implies that the DNA entirely unwrapped from the H2A-H2B heterodimer and reflects a weakened DNA-histone binding^57^ leading to a spontaneous site exposure^58,59^.

The distribution of *θ* angles showed a slight shift of the peak position from 41° to 50° in the presence of H1 (Extended Data Fig. 5a, **row 4 and 6, left**). However, its two components display more significant changes. First, the major peak of *θ*_∥_ shifts from −7° to −48° with H1, which was similar to the shift observed in 50 mM Na^+^ (from −7° to −42°) (Extended Data Fig. 5a, **row 4-6, middle**). Second, a minor peak of *θ*_∥_ emerged at 136° in 28% of the NCPs, adopting an alternative “wide-open arm” conformation (Extended Data Fig. 5a, **red arrow**). Third, the major peak of *θ*_*⊥*_ showed the DNA linkers adopting a more parallel arrangement when viewed from the side of the NCP in the presence of H1 (from 35° to 6°), similar to the change caused by the increase of Na^+^ (from 35° to 19°) (Extended Data Fig. 5a, **row 4-6, right**). Finally, a second peak of *θ*_*⊥*_ at 152° suggests the co-existence of alternative conformations (Extended Data Fig. 5a, **green arrow**). Classification analysis (Extended Data Fig. 9b, **orange arrows**) confirmed that these minor peaks mainly resulted from DNA unwrapping around the second NCP (Fig. 4e, II).

The *α* and *β* angles show the major population of DNA linkers turned toward each other in the presence of H1, likely due to the “triangular-shape” formed between the NCP and its two linkers (Fig. 4e, **red triangles**) (*i.e.* the major and minor peak of *α* decrease from 32° to 27° and −8° to −16°, respectively, while the *β* peak decreases from −9° to −1°, Fig. 4f and Extended Data Fig. 6c-d, **row 4 and 6**). These changes were again, similar to those induced by 50 mM Na^+^ (Extended Data Fig. 6c-d, **row 5**). Interestingly, these two different conditions modulate the DNA linkers dynamics on the NCP in a similar manner even though one involves electrostatics screening and the other protein binding^10,25^.

Characterization of the nearest-neighbor distance of NCPs shows that the major peak of consecutive NCPs was slightly decreased by H1 (from 237 Å to 205 Å), an effect also seen by the increase of Na^+^ (from 237 Å to 218 Å) (Extended Data Fig. 7b, **row 3-5, left**). The second peak appearing at 313 Å (Fig. 4g, **left)** likely reflects co-existing distal arm invading conformations (Fig. 4e), in which the linker **between i** and **i+1** NCPs was extended due to the DNA unwrapping. Meanwhile, the major peak depicting the distance between **i** and **i+2** NCPs only decreases from 236 Å to 213 Å compared to the reduction caused by 50 mM Na^+^ (from 236 Å to 114 Å) (Extended Data Fig. 7b, **row 4-5, middle**), and the second peak moved even further to a distance of 453 Å, which indicates a volume expansion of the array instead of a compaction. This result is also confirmed by the distance between the *i* and *i+3* NCPs (Extended Data Fig. 7b, **row 4-5, right**).

The analysis of NCP dihedral angles showed that H1 did not induce a preferred orientation among NCP discs compared to those acquired under 50 mM Na^+^ (Extended Data Fig. 7d-e, **row 5**). Combined with the angle/distance analysis, these observations suggest that H1 can only partially regulate the arrays conformation, such as by changing the direction of DNA linkers on NCPs, but not the interaction among NCPs. The reason may be as follows: the presence of H1 bridges locks the two DNA linkers/arms of an NCP, yielding a similar outcome as high salt concentrations by drawing them closer together. However, H1 is not able to reduce the electrostatic repulsion among the charged NCPs due to the high ionic strength via charge screening. As a result, H1 alone at low-salt conditions cannot induce the full compaction of the array. The appearance of conformations in which the upstream DNA arm of the first nucleosome invades and wraps around the second nucleosome (Fig. 4e, **panel II**) may reflect the fact that, at low ionic strength, NCPs are kept separated at “preferred” distances leading in some cases to partial unwrapping of the DNA, which in turns permits the wrapping of “foreign DNA”. It also leads to additional DNA junctions that provide other possible binding sites for H1 (Fig. 4e, **II and III, black arrow**), which could further stabilize the system. It is worth noting that the invasion of the second NCP is the dominant conformation observed, compared to those of the third and fourth NCPs, which yield two dinucleosome regions separated by a longer linker (Fig. 4e, **II, red-dashed line**). This experiment suggests H1 reduced the freedom range of two DNA linkers as reported ^60^, while increasing the connectivity among the NCPs but not significantly reducing the overall diameter.

### Modelling the effect of linker DNA orientation and dynamics on chromatin morphology

**Extended Data Fig. 10:**
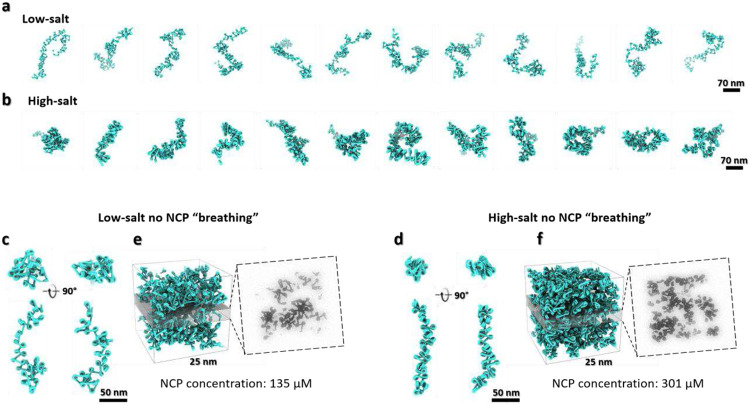
**a,b,** Twelve representative simulated chromatin fibers containing 100 NCPs in 5 (**a**) and 50 (**b**) mM Na^+^, respectively. **c,d**, In silico assembly of chromatin fibers containing 100 NCPs using only the α, β angle distribution from cryo-EM experiment in 5 (**c**) and 50 (**d**) mM Na^+^ conditions (only the first 40 NCPs were shown). The NCP “breathing” is eliminated by confining the DNA unwrapping level to a fixed number equal to the mean of the corresponding distribution. **e,f**, Simulation of a chromatin domain organization (left panel) and central slice representation (right panel) after eliminating NCP “breathing” in 5 (**e**) and 50 (**f**) mM Na^+^ conditions, respectively.

**Extended Data Fig. 11:**
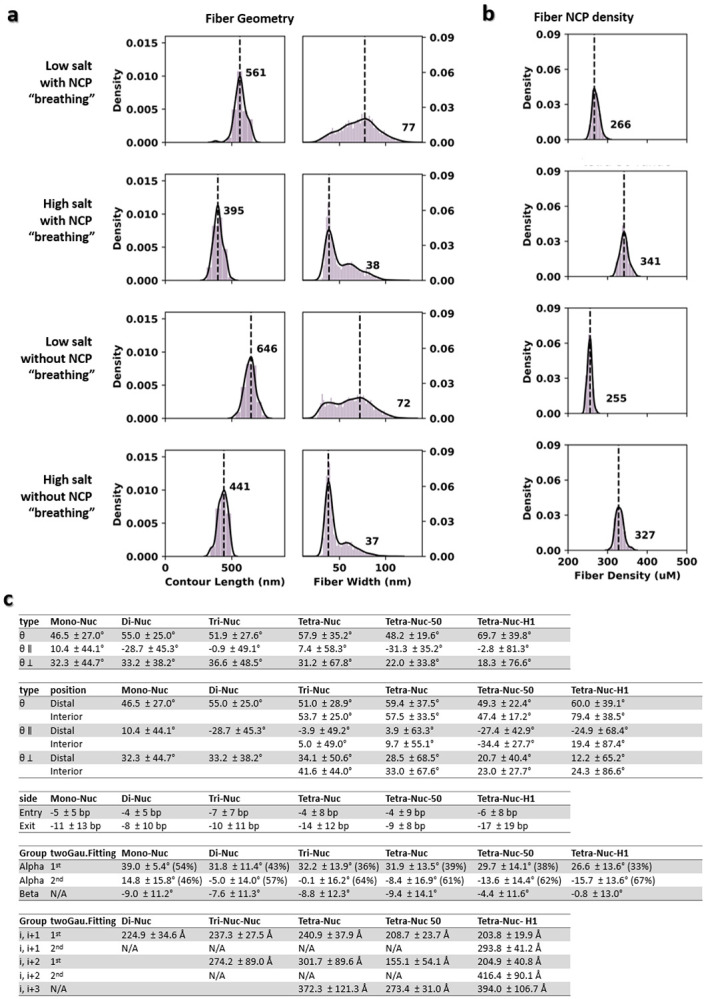
Distribution of the in-silico hecta-nucleosome array fiber length, width, and NCP density and the statistics of the experimentally measured array parameters. **a**, Histograms of the measured contour length (left columns) and width (right column) of the simulated nucleosome array fiber with 100 NCP units. 100 simulated array fibers are generated for each category (row 1 through 4) to calculate the statistics **b**, Distribution of the NCP density measured from the corresponding fibers in (**a**). The density is calculated by dividing the total number of NCP by the corresponding fiber volume. **c**, The statistics used to generated the simulated hecta-nucleosome array fibers in (**a** and **b**) extracted from the experimentally measured array parameters.

**Fig. 5:**
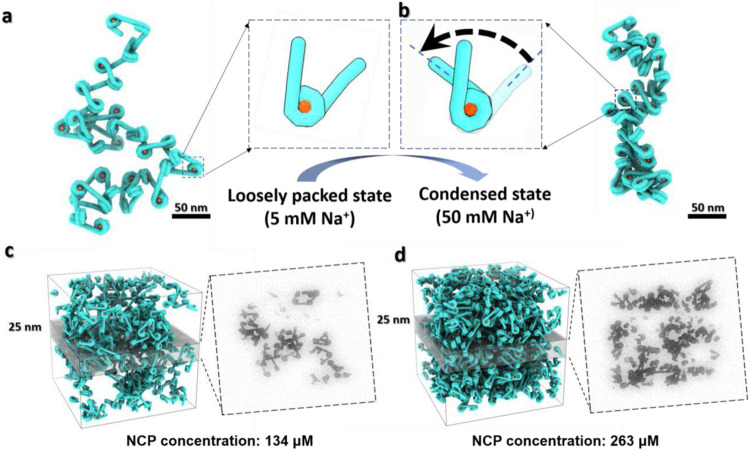
Modeling chromatin fiber conformational regulation mechanism by linker DNA angles. **a,b**, In silico assembly of a long chromatin fiber by connecting 100 NCPs following the α, β angle distribution and the NCP unwrapping distribution acquired from the experimental statistics of the tetranucleosome arrays in 5 mM (**a**) and 50 mM (**b**) Na^+^, respectively (only the first 40 NCPs are shown). **c,d**, Simulation of chromatin domain organization constructed by sequentially fitting 100 NCP fibers generated from (a) and (b), respectively, within a 200-nm cubic volume. The nearest inter-array spacing follows the *i*, *i+3* core-to-core distance distributions measured from the tetranucleosome arrays at the corresponding experimental conditions. A central slice of 25 nm thickness is cropped from each cubic volume colored in gray.

To validate whether the angle change between two DNA linkers can really modify the large-scale morphology and density of chromatin fibers, hecta-nucleosome arrays were computationally generated based on the obtained statistics. The analysis of simulated hecta-nucleosomes at 5 mM Na^+^ showed curvy irregular fibers (Fig. 5a and Extended Data Fig. 10a) with an average length and width of 561 ± 44 nm and 77 ± 19 nm, respectively (Extended Data Fig. 11a, **row 1**). By using the parameters obtained at 50 mM Na^+^, both the chromatin length and width were reduced to 395 ± 35 nm and 38 ± 16 nm, respectively (Extended Data Fig. 11a, **row 2**), producing irregular/disorganized and condensed “30 nm fibers” (Fig. 5b and Extended Data Fig. 10b) as observed experimentally^61,62^.

To simulate possible chromatin condensates, the generated fibers were inserted into a 200 nm cube with two different approaches: “seamless assembling” *vs* “random docking” (for details see methods). Results showed that the estimated density of condensates were between 134 - 266 μM at low-salt conditions. These values were nearly doubled (263 - 341 μM) at 50 mM Na^+^ conditions (Fig. 5c-d and Extended Data Fig. 11b, **row 1-2**). These chromatin densities are within the experimentally measured range of 80-520 μM *in vivo* ^30^ and ~340 μM *in vitro* ^21,28^.

The 30-nm fibers generated in this simulation were not often observed in cryo-EM imaging^17,63^ or cryo-ET cell sectioning slices^32,46^. One possible explanation for this discrepancy is the low probability of capturing fibers lying exactly on the plane of the thin slice cell section, especially when the latter approaches a thickness of 30 nm^12,46^. Slicing through the simulated condensates across the z-axis with a 25 nm-thick slab greatly reduces the fiber pattern and replaces them with more irregular structures (Fig. 5c-d **left panel**).

Because NCP “breathing” dynamics plays a critical role in biologically relevant DNA processes, such as enabling restriction enzyme access to the inner wrapped DNA target site^58^ and nucleosome translocation without the participation of chromatin remodelers^64^, we repeated the above simulations by explicitly preventing DNA unwrapping. The resulting simulations increased the length of the chromatin fibers by 11-13% (from 561 nm to 646 nm at low-salt, and from 395 nm to 441 nm at high-salt) (Extended Data Fig. 10c-d and 11a-b, **row 3-4**), but did not induce an obvious change of the condensate’s density in either salt conditions (Extended Data Fig. 10e-f), suggesting that breathing does not increase enzyme accessibility to the interior of the condensates. Instead, the DNA linker angles of the arrays are found to have a major effect on the density of condensates and the morphology of chromatin.

Unlike conventional averaging methods, in which a population of particles possessing homogeneous structural identity is selected from a large and diverse pool of particles in thermodynamic equilibrium, the individual-molecule approach of cryo-ET provides continuous particle conformational distributions by randomly sampling the system. In this case, the probability (*p_i_*) of finding a molecule in an energy level (*U_i_*) is proportional to *e*^−U_i_/k_b_T^ based on the Boltzmann distribution, where *k*_b_ is the Boltzmann constant and *T* is the temperature. Thus, the energy difference (Δ*U_ij_* = *U_i_* – *U_j_*) between two energy states can be calculated from Δ*U_ij_* = *U_i_* – *U_j_* = −*ln*(*p_i_/p_j_*) × *k_b_T*. In this study, we observe two peaks of *α* angles in all the molecular populations studied (Extended Data Fig. 11c), which correspond to two energy states. The calculated energy difference between the relatively opened and closed DNA arm conformation were 0.17 *k_b_T* (given the corresponding populations of 54% *vs.* 46%), 0.27 *k_b_T* (43% *vs*. 57%), 0.56 *k_b_T* (36% *vs*. 64%) and 0.43 *k_b_T* (39% *vs*. 61%) for mono-, di-, tri-, and Tetranucleosome arrays, respectively. However, the energy differences changed to 0.48 *k_b_T* at 50 mM Na^+^ (38% *vs*. 62%) and 0.69 *k_b_T* in the presence of H1 (33% *vs.* 67%). Since the bending energy of wrapped nucleosomal DNA is in a range of 0.34 to 0.46 *k_b_T*/bp^65^, the transition between the two conformations requires only the bending of one or two DNA bp per nucleosome, which results in the observed large-scale array conformational changes. These simulations confirmed the angle between two DNA linkers plays a key role in regulating the overall morphology and density of chromatin fibers.

## Conclusions

The structural dynamics and conformational changes of loosely packed nucleosome arrays are critical to our understanding of transcriptionally active chromatin architecture prior to the formation of compacted nucleosomal condensates. To gain insights into the nucleosome array structure in the active state and their conformational change in response to chemical and biological factors, we have determined the conformation for each individual nucleosome array particle (without averaging) by cryo-ET. We found that an increased ionic strength reduced the angle between the DNA linker and the NCP, which resulted in a decreased size and increased density of the asymmetric tetranucleosome array unit. In comparison, H1 likewise changes the dynamics of the array but builds alternative nucleosomal networks. The surface physical properties of NCP, such as polarization and hydrophobicity, may be changed by altering the geometry of the nucleosome array, which may then strengthen the intermolecular interaction for the formation of a spinodal structures. The regulation of gene expression followed the development of the spinodal structure into compacted spherical condensates, which may serve as the underlying mechanism of how chromatin mediates the change from interphase to metaphase structures. The new insight into these conformational changes prior to the nucleosome arrays intermolecular interactions extends our knowledge to understand the regulation mechanism of nucleosome arrays via phase separation.

## Materials and Methods

### Expression and purification of histones

Vectors containing the genes of *Xenopus* laevis histones H2A, H2B, H3, and H4 under the control of a T7 promoter were expressed in *E. coli* BL21(DE3) and purified as previously described ^66^. For each histone, a single *E. coli* BL21 (DE3) colony was grown at 37°C in LB media supplemented with 100 μg/uL ampicillin and 25 μg/uL chloramphenicol to an OD_600_ of ~0.6, and histone expressions were induced by adding 1mM Isopropyl b-D-1-thiogalactopyranoside (IPTG) to the cell culture. After 3 h, the cell culture was centrifuged at 7,000 × g for 30 min, and the cell pellet was suspended in ice-cold wash buffer (50 mM Tris-HCl pH 7.5; 100 mM NaCl; 1 mM EDTA; 5 mM 2-mercaptoethanol (BME); 1% Triton X-100 [w/v]; and protease inhibitors, Roche). This procedure was repeated twice to wash away LB medium. The cell pellets were suspended in 4 volumes of wash buffer, flash freezing with liquid nitrogen, and stored at −80°C for later use. To purify the inclusion bodies, suspended cell pellets were thawed and sonicated on ice seven times with 20-s bursts at 7.0 W with a Misonix 2000 sonicator. The lysate was centrifuged at 30,000 x g for 1 h at 4°C, and the pellets were rinsed by suspension with wash buffer. This procedure was repeated three times. To remove Triton X-100, pellets were rinsed by suspension using washing buffer without the detergent. Suspended pellet was centrifuged at 30,000 x g for 1 h at 4°C and this procedure was repeated three times. Inclusion bodies were then solubilized in 20 mM Tris-HCl pH 7.5; 8 M Urea; 1 mM EDTA; 10 mM DTT, and purified by anion and cation exchange. Purification was checked by 15% SDS-PAGE and fractions containing pure histones were pooled and dialyzed against 1 L of 10 mM Tris pH 8.0. Histones were centrifuged to remove aggregates, concentrated (~10 mg/mL) using a 10K Amicon Ultra-15, lyophilized, and stored at −80°C for later use.

### Histone octamer purification

The reconstitution of the *X. laevis* histone octamer was performed as previously described (Wittmeyer). Briefly, lyophilized histones were solubilized with unfolding buffer (20 mM Tris-HCl pH 7.5; 7 M guanidine hydrochloride; 10 mM DTT), and combined at a H2A:H2B:H3:H4 ratio of 1.2:1.2:1:1. The volume was adjusted to a total histone concentration of 1 mg/mL and it was incubated with mixing for 3 h at room temperature. Solubilized histones were dialyzed four times for 12 hours each against 1 L of refolding buffer (10 mM Tris-HCl pH 8.0; 2 M NaCl; 1 mM EDTA; 5 mM DTT) using a 3.5 kDa dialysis membrane at 4°C. Refolded histone solution was centrifuged at 100,000 x g for 30 min to remove aggregates, concentrated by centrifugation to ~0.5 mL using 10 kDa Ultra-15 (Milipore), and loaded onto Superdex 200 Increase 10/300 GL (Cytiva) previously equilibrated with refolding buffer. Fractions were checked by 15% SDS-PAGE and the gel was stained with AcquaStain protein staining (Bulldog Bio). Fractions containing three bands (corresponding to H3, H2A/H2B, which, because of their similar size they are not resolved in the gel, and H4) and in equimolar quantities (based on the intensity of the three bands) were pooled, concentrated to ~8-10 mg/mL using 30 kDa Ultra-15 (Milipore), aliquoted, and flash-frozen with liquid nitrogen, and stored at −80°C for subsequent use. The synthesis of the H2A-H2B dimer followed the same procedure utilized for octamer reconstitution, with H2A and H2B combined at a ratio of 1:1.

### Synthesis of DNA templates

The mono-, di, tri-, and tetranucleosome DNA templates consists of one, two, three, and four repeats of the 601-nucleosome positioning sequence (NPS), respectively. Each template is flanked to the left by 200 bp DNA and to the right by 100 bp DNA, and the 601 NPS in di-, tri-, and tetranucleosome templates are separated by a 40 bp linker length. Mononucleosome DNA templates were amplified by PCR from the pGEM-3Z/601 plasmid (addgene) and cloned back into the PGEM 601 vector using primers containing the restriction recognition site for the *BsaI* enzyme (NEB). Di-, tri-, and tetranucleosome DNA templates were synthetized by ligation of two, three, or four PCR products containing one 601 NPS each. The PCR products were amplified using the pGEM-3Z/601 plasmid (Addgene) and primers containing the restriction recognition sites for enzymes *BsaI* and *BbsI* (NEB). In our design, the di-, tri-, and tetranucleosome DNA are flanked by *BsaI* restriction sites, and the *BbsI* sites were used to ligate the two, three, or four PCR products, respectively. To generate the full templates, the PCR products were digested first with *BbsI* and ligated at an equimolar ratio using *E. coli* DNA ligase (NEB). The longest product of each ligation, corresponding to the di-, tri, or tetranucleosome DNA, were purified using 0.8% agarose gels, digested using the BsaI enzyme, and cloned into *BsaI*-restricted pGEM-3Z/601 plasmid using T4 DNA ligase (NEB). Ligation product was transformed into *E. coli* DH5*α* for plasmid extraction by miniprep. Each ligation product was checked by DNA sequencing. Plasmid containing the mono-, di-, tri-, and tetranucleosome templates were grown in *dam*^−^/*dcm*^−^ *E. coli* (NEB), purified by maxiprep, and excised by restriction with *BsaI*. Mononucleosome DNA template was purified from the vector backbone by 5% preparative acrylamide electrophoresis using a Model 491 Prep Cell (Bio-Rad) electroelution system. Di-, tri-, and tetranucleosome templates were purified by 4% preparative acrylamide electrophoresis.

### Nucleosome assembly and purification

*X. laevis* histone octamer and mono-, di-, tri, and tetranucleosome DNA templates were combined at a ratio of 1:1.2, respectively, in high-salt buffers (10 mM Tris-Cl pH 8.0; 2 M NaCl; 1 mM EDTA; 0.5 mM DTT; and 1 mM PMSF) at a final DNA concentration of 100 ng/uL. In the case of the arrays, H2A-H2B heterodimer dimer was also incorporated at a molar ratio of 0.2 compared to the octamer. Assembly solutions were dialyzed using a 3.5 kDa dialysis membrane at 4°C against 500 mL of high-salt buffer for 1 h at 4°C, followed by a 36-hour lineal gradient dialysis against 2 L of low-salt buffer (10 mM Tris-Cl pH 8.0; 1 mM EDTA; 0.5 mM DTT; and 1 mM PMSF) with continuous stirring. A final dialysis of 3 h was performed against 500 mL of low salt buffer. Nucleosome reconstitutions were checked by 4% acrylamide and 0.2X TBE buffer (Tris-borate-EDTA) native electrophoresis. Mononucleosomes were purified from hexasomes and bare DNA by 4% preparative acrylamide (59:1 acrylamide:bisacrylamide) electrophoresis using the 491 Prep Cell (Bio-Rad). Di-, tri-, and tetranucleosomes were purified by 10-40% lineal sucrose gradient (20 mM HEPES-NaOH pH 7.5; 1 mM EDTA; 1 mM DTT) and centrifuged for 16 h at 38,000 rpm at 4°C, using an ultracentrifuge Beckman Optima MAX-XP with the rotor MLS-50 (Beckmann), as previously described (Mol cell paper reference).

### TEM specimen preparation

The cryo-EM specimens were prepared following the procedure described before^37^. Briefly, an aliquot (~3 μl) of nucleosome array sample ~400 nM was placed onto the 200 mesh Quantifoil copper grid (Q210CR-06, Electron Microscopy Sciences) that had been glow-discharged for ~15 s by (PELCO easiGlow^™^ Glow Discharge Cleaning System) for 15 seconds. After incubating for ~10 sec, the grid was flash-frozen in liquid ethane at ~90% humidity and 4 °C with a Leica EM GP rapid-plunging device (Leica, Buffalo Grove, IL, USA) after being blotted with filter paper with a controlled blotting time (2 s). The flash-frozen grids were transferred into liquid nitrogen for storage.

The NS-EM specimens of nucleosome array sample were prepared using the optimized negative-staining protocol (OpNS) as described ^67^. In brief, the samples were diluted to ~20 nM with sample buffer. An aliquot (~4 μL) of diluted sample was placed on an ultra-thin carbon-coated 200-mesh copper grid (CF200-Cu-UL, Electron Microscopy Sciences, Hatfield, PA, USA) that had been glow-discharged for ~15 s. After ~1 min incubation, the excess solution on the grid was blotted with filter paper. The grid was then washed with water and stained with 1% (w/v) uranyl formate (UF) before air-drying with nitrogen.

### TEM data acquisition

Cryo-EM specimens were screened by a Titan Krios (FEI) transmission electron microscope operated at 300 kV high tension with a Gatan energy filter. The untitled cryo-EM micrographs were acquired with a Volta phase plate under defocus at ~2.5 μm using a Gatan K2 Summit direct electron detection camera under a magnification of ~81 kx (each pixel of the micrographs corresponds to ~0.9 Å in specimens). The particles with a box size of 800 × 800 pixel will be used to display the morphology. Cryo-EM tilt image series of the samples were collected from −60° to +60° at 3° increments on a Titan Krios G2/G3TEM equipped with a Gatan energy filter and a K2 Summit direct electron detection camera operated under 300kV high tension. During data acquisition, the SerialEM^68^ software was used to automatically track the specimen and maintain defocus at ~2.5 – 3.0 μm. The acquired tilt image series at magnification of ~81/50 kx (each pixel corresponds to 1.46/1.47 Å for K2/K3 camera) represents a total dose of ~102 - 183 e/Å^2^. For each tilt angle, a total number of ~8 frames were collected under the exposure of 0.25 s per frame.

NS-EM specimens were screened by using a Zeiss Libra 120 Plus TEM (Carl Zeiss NTS) operated at 120 kV high tension with a 10-20 eV energy filter. The OpNS micrographs were acquired under defocus at ~0.6 μm using a Gatan UltraScan 4K × 4K CCD under a magnification of 125 kx and 160 kx (each pixel of the micrographs corresponds to ~0.94 Å and ~0.74 Å in specimens respectively). Tilt image series of mononucleosome samples were collected from −60° to +60° in 3° increments using a Zeiss Libra 120 Plus TEM (Carl Zeiss NTS) equipped with an in-column energy filter and a Gatan UltraScan 4K X 4K CCD. During data acquisition, the Gatan tomography module (Gatan Inc., Pleasanton, CA, USA) operated in Advanced Tomography mode was used to track the specimen and maintain defocus at ~2.0 μm. The acquired tilt image series at magnification of 50 kx (each pixel corresponds to 0.24 nm) represents a total dose of ~60 e^−^/Å^2^.

### Image preprocessing

The motion of the cryo-ET frames was corrected by MotionCor2^69^. To reduce the cryo-ET image noise, we followed a machine learning method (NOISE2NOISE, T2T method) as described^38^. The contrast transfer function (CTF) of both cryo-ET and NS-ET tilt series was measured by using ctffind3 software^70^ and the phase and amplitude were corrected by SPIDER^71^, GCTF^72^ and TOMOCTF^73^ after the X-ray speckles were removed. The tilt series were initially aligned by using IMOD^74^. By using boxer from EMAN software^75^, a box of 256 × 256 pixels was used to select the particles of nucleosome from the ~113 micrographs imaged under 125 k× magnification, while a box of 200 × 200 pixels was used to select the segments of the upstream (or entry) and downstream (or exit) DNAs from the ~150 micrographs imaged under 160 k× magnification. All NS-EM particles were masked using a round mask generated from SPIDER software after a Gaussian high-pass filtering. The reference-free class averages of particles were obtained using refine2d (EMAN software) based on 30,540 particles of DNA segment and 13,029 particles of NCPs.

### Individual particle electron tomography (IPET) 3D reconstruction

The tilt series of each of the targeted particles were semi-automatically tracked and then windowed in square windows of ~ 1,000 × 1,000-pixel size using IPET software^37^, before being binned four to five times to reduce computation time in subsequent reconstructions. Following the pipeline of IPET reconstruction^37^, a local tilt series images containing a single nucleosome particle was extracted from the IMOD-aligned full-size tilt series to perform focused ET reconstruction (FETR), which can eliminate the effects from the large image distortion^37^. Briefly, an *ab initio* 3D density map was directly back-projected in Fourier space and served as an initial model. The refinement was then iteratively invoked to translationally align each tilted particle image to the computed projection. During the refinement, automatically generated Gaussian low-pass filters, soft-boundary circular masks and loose particle-shaped masks were sequentially applied to the tilt images and references to increase the alignment accuracy^37^. An improved model was then reconstructed based upon the refined alignment at the end of each refinement iteration. The 3D map was then reconstructed by back-projection of the filtered and masked particle tilt series. The final back projection was performed in Fourier space without weighting. To reduce the artifact caused by the limited tilt angle range, the final 3D map was submitted for a published missing-wedge correction method by LoTToR ^39^, in which the low resolution mask used was generated by the Model-Based Iterative Reconstruction (MBIR) method^76^. All final density maps were low-pass filtered to 4.5 nm using EMAN^75^ and displayed using UCSF Chimera^77^.

### Estimation of the reconstruction resolution

The resolution for the reconstructions were estimated by two methods. i) Data-to-Data based analysis: the Fourier Shell Correlation (FSC) was calculated between two independently reconstructed 3D maps, in which each map was based on one-half of the tilt-series (split by even and odd tilt index) after particle alignment refinement of the IPET^37^. The frequencies at which the FSC curve first falls to values of 0.5 and 0.147 were used to represent the reconstruction resolution. Notably, the resolution estimated by this method could be severely under-estimated since the reconstruction from one half of the tilt-series significantly reduced the quality of the map compared to the final reconstruction. ii) Data-to-Model based analysis: the FSC curve between the final IPET reconstruction and the density map converted from the corresponding fitting model was calculated. The frequencies at which the FSC curve fell below 0.5 was used to estimate the resolution. The density map of the fitting model was generated by pdb2mrc in EMAN software^75^.

### Modeling the structure of nucleosome arrays

To build a structural model for the reconstructed map of each individual nucleosome array particle, the pathways for the two flanking DNA arms were initially traced by sampling a group of 3D points located at the high-density loci of the map followed by sorting their order into a points list. Then, by fitting the crystal structure of Xenopus laevis NCP into the discoidal-shaped high-density region, the DNA pathways with the NCP were also defined and converted to a list of 3D points. These points series from different models were merged together after removing adjacent clashes and then fitted with a smooth quadratic Bezier curve followed by conversion into DNA model by using GraphiteLifeExplorer^78^. The total length of the DNA used to thread the model matched with our designed DNA construct, *i.e.* 456, 632, 818, 1008 for mono-, di-, tri- and tetranucleosome arrays, respectively. Notice that for some of the maps, a small portion of DNA densities (~10%) was missing near the middle portion of DNA arms (which may be due the orientation of DNA segments aligned near-perfectly perpendicular to the beam direction, resulting the lowest image contrast of the DNA). Fortunately, those missing portions were small and did not prevent the DNA model fitting, which can be circumvented by interpolating surrounding density at those loci. To further refine the model, the Molecular Dynamics Flexible Fitting (MDFF)^40^ was applied to energy minimize the model under a force gradient created by the electron density map.

### Evaluating the fitting models

To evaluate the self-consistency of the above fitted models, the correlation between the measured DNA linker length between two consecutive NCPs (named as *L*(*n*)) and the estimated DNA unwrapping level between the same NCPs were calculated. The *L*(*n*) was acquired from the experimental data, in which distance between the DNA exit position of the nth NCP and the entry position of the *(n+1)^th^* NCP from the density map were measured. The DNA unwrapping level was estimated from the fitted model, in which the unwrapping angles from the exit side of nth NCP and entry side of the (n+1)^th^ NCP were added together (*θ*(*n*) = *θ*_*ex*_(*n*) + *θ*_*en*_(*n* + 1)). The Pearson correlation coefficient, r=−0.9 was calculated from the linear regression fitting of the unwrapping length *L*(*n*) against the angle *θ*(*n*) using the statsmodels package in Python. The off-template NCPs (binding to the 100 or 200-bp distal arms region) are removed from the following statistical analysis.

### Defining the entry and exit linker DNA origins on the NCP

By using the fitted nucleosome array models, the entry and exit DNA arm origins on the NCP can be estimated with the following procedure. The DNA portion of the fitted nucleosome array model was first converted into a list of 3D points (series m) by averaging the coordinates of C1 atoms of each base-pair. After aligning the nucleosome crystal structure to the fitted array model at its nth NCP region, a points list (series n) along the wrapped DNA from the nucleosome crystal structure was also generated by the same method. By comparing the two points lists, the overlapped region on the fitted DNA model can be identified if any points from the series n were found within its 8 Å radius of series m. The 8 Å criterion was chosen based on that, when two DNA helical centers separated away from each other for over one third of the DNA diameter (~24 Å), the separation of two aligned DNAs can be distinguished. The base pair indexes at the two distal ends of this overlapped region were used to define the entry and exit DNA linker arm origin along the array. The total number of the base-pairs between the entry and exit position were used to calculate the length of wrapped DNA on the histone surface.

### Measuring the NCP wrapping dynamics on histone surface

By measuring the position and length of the wrapped DNA on each histone surface, the DNA unwrapping footprint along the array can be quantified as follows. For a fitted array model, a binary score was assigned to each DNA base pair along the DNA sequence depending on its contact with the histone octamer (“1” for contact if the atoms of the base pair fall in a 4 Å distance with any histone octamer atoms and “0” for non-contact). By averaging the scores of the *i^th^* base pair within same type of fitted array models, the mean score distributions along the DNA template (from upstream to downstream) for a mono-, di-, tri-, and tetranucleosome array were calculated separately. The score distributions around the regions contains the designed 146-bp Widom 601 position sequence^79^ reflects the probability of finding DNA unwrapping events. The difference of the score distribution for the entry and exit side of each NCP loading position reflects the asymmetry property of the DNA sequence in binding to histone octamers and the dynamic relationship between DNA unwrapping and equilibrium assembling, such as transition states among the tetrasome, hexsome and full octasome^80^.

### Defining the entry and exit linker DNA vectors on the NCP

Due to the fact that the 20-bp DNA segment (~6.8 nm) is relatively stiff compared to the persistence length of DNA ~50 nm, the base pairs residing at the entry side DNA arm origin and its 20-bp upstream were used to define the start and end-point of a vector, respectively (based on their helical center coordinates). This vector was used to represent the entry DNA arm pointing direction. Similarity, the base pairs residing at the exit side DNA arm origin and its 20-bp downstream were used to define the exit DNA arm vector.

### Measuring the angle *θ*, wrapping angle *α* and bending angle *β* of the DNA arm vectors

The angles of the DNA linker arms that extended from the discoidal-shaped NCP surface were measured by the following steps. i) Defining the X-, Y-, and Z-axes of each NCP model. The Z-axis was defined along the helical axis of the wrapping DNA measured from its rotational symmetry. The Y-axis was defined by the dyad axis of the NCP. The cross-point of these two axes was used as the center of NCP and defined the X-axis, where it simultaneously passed the NCP center and was perpendicular to both Z- and Y-axis. ii) Defining the relative DNA arm angle θ. θ measured the angle between the previously defined entry and exit DNA linker arm vectors on the same NCP. Because DNA arm conformational states (“open and close”) must be defined relative to the NCP, the projections of the θ angle on the NCP discoidal plane (X-Y plane) and its perpendicular plane (Y-Z plane) were measured as θ∥ and θ*⊥*, respectively. iii) Defining the in-plane wrapping angle *α* of an NCP arm vector. *α* calculated the angle formed by two vectors within the X-Y plane. One vector is the projection of the NCP linker arm vector on the X-Y plane, and the other vector is defined by the tangential direction of the discoidal-shaped projection of NCP on X-Y plane, which crosses the origin of the corresponding NCP linker arm. iv) Defining the out-of-plane bending angle *β* of an NCP arm vector. *β* calculated the angle formed by the NCP arm vector and the X-Y plane. The measured angle distribution was fitted with either one gaussian or two gaussians with the sklearn.mixture.Gaussian Mixture package.

### Measuring the intra-array NCP core-core distances and plane-plane angles

To quantitatively define the spatial relationship among the NCPs from different types of nucleosome arrays, the core-core distances and plane-plane angles between each pair of NCPs were measured. The core-core distance, *D*(*n*, *n* + *m*), measured the distance from the center of the nth to the (n+m)^*th*^ NCPs. The core-core angle, *φ*(*n*, *n* + *m*), measured the dihedral angle between the discoidal planes (X-Y planes) of the nth and (*n*+*m*)^*th*^ NCPs. The histograms were fitted by a Kernel Density Estimation (KDE) function^81^. The dihedral angle distributions were compared with a sine function, which represents a distribution of orientations of two random planes (the angle θ between their normal directions), which in turn are equivalent to the probability of finding a point on the unit hemisphere contained in a differential ring-shape area:

$$P(\theta )d\theta =\frac{2\pi [R\;sin(\theta )]Rd\theta }{2\pi R^{2}}=sin(\theta )d\theta$$

### Insilco-construction of the chromatin-like higher order structure

The distribution of the wrapping and bending angles relative to the NCP were used to build longer in silico nucleosome array fibers. Each longer array fiber containing 100 NCPs was generated by randomly connecting nucleosome model units. To be specific, due to the large number of atoms within the NCP (>12k) and the fact that the length of 40-bp DNA (~14nm) is much smaller than the persistence length of DNA (~50 nm), NCP units were coarse grained where the histone octamer and linker DNA arms were treated as spheres and straight lines, respectively. Based on the distribution of the measured mean and standard deviation of the bending and wrapping angles (*i.e.* *α* and *β*) for the tetranucleosome array at low salt and high salt condition, two vectors in a 20-bp length followed the same angle distributions relative to the NCP were randomly generated and used to represent the entry and exit DNA linker arms by using VMD software^82^. By repeating this process, a pool of NCP units with various linker arm conformations but following the same spatial distribution to the experimental data were prepared. To assemble the NCPs units into a fiber, randomly selected NCPs from the pool were sequentially connected together based on the following procedures: i) The exit side 20-bp linker for the ith NCP was linearly connected to the entry side 20-bp linker for the i+1th NCP, ii) The 40-bp linker causing ~70° left-handed DNA rotation has been considered^83^, in which the *(i+1)^th^* NCP unit was rotated along the entry side linker vector after connecting to its previous NCP. iii) if the *(i+1)^th^* NCP was identified to clash with any of the previous NCPs, a new NCP will be randomly selected from the pool and resembled onto the ith NCP by repeating step i through iii. In order to simulate more realistic dynamics of the nucleosome fibers, the unwrapping events were also considered into the system. The origin of the entry and exit DNA linker vectors on the helical NCP track were also varied based on the experimentally measured distribution when constructing the pool of the NCP units.

### Insilco-construction of the nucleosome condensates

To reconstitute the chromatin-like higher order structures, a pool of 100 of the above constructed fibers was prepared by using low- and high-salt angle and unwrapping parameters. The assembling of these fibers followed two approaches: “seamless assembling” vs “random docking”. In the first approach, the condensates were assembled with no space between array fibers. In this case, the density of the condensate is simply equal to the average density of the above simulated chromatin fibers, which yielded the upper bound to estimate the condensate density. In the second case, all fibers were treated as rigid-bodies and then sequentially fitted into a spherical volume density in a diameter of 200 nm given an inter-array distance. This distance was estimated by the experimentally measured i and i+2 NCPs distance, which mostly reflects the equilibrium NCP interaction distance within an array. To do so, array fibers were converted into low-pass filtered density maps with specific threshold cutoff values that followed the distribution of the measured i and i+2 NCPs distance. The produced fiber density maps were randomly fitted into the spherical volume by using the Chimera sequential fitting function without clashing. The central cubic volume in 20 nm length was cropped out from the generated large condensate for the final representation. The measured density from the second approach was used as the lower-bound to estimate the condensate density.

### Classification of the nucleosome array conformations

To identify some of the low energy conformational state of the nucleosome array, which should be observed more often than others based on Boltzmann distribution, nucleosome arrays were classified based on their structural similarity. For each type of the array, 30 to 40 conformations were obtained and subjected to a pair-wise alignment through minimization of the root-mean-square deviation (RMSD) between each pair of models. Based on the values of the constructed distance matrix evaluated by RMSD, the array conformations were sorted and classified with an optimization algorithm that minimized the tree spanning (method option = ‘single’) using the hierarchical clustering in Scikit-Learn of Python package^84^. The final result was displayed in a dendrogram produced by the scipy.cluster.hierarchy package.

### Visualization of the structure dynamics of the nucleosome array

The pseudo dynamics of nucleosome array was simulated by morphing through array conformations in an order followed by the hierarchical clustering. The morphing begins with the structures at the lowest branch of the dendrogram and stops at the highest branch. By using Targeted Molecular Dynamics simulation (TMD) of the NAMD2, the coarse-grained structure was steered from one conformation toward to another under the amber SIRAH force field. The moving forces used in the TMD were pre-calculated based on the distances of the corresponding CG model atoms. After 1,000,000 to 4,000,000 steps (corresponding to a total time of 20 ns to 80 ns, with each step of 20 fs) at 298 K temperature within the implicit solvent, the simulation was terminated when the real-time RMSD fell below 3 Å. Among these morphing structure pairs, ~10% of them were eliminated due to the DNA intertwining with each other. The morphing movie was also displayed with the same order as above from the most populated structure states to the rarest states.

### Analysis of in-silico nucleosome array fiber stiffness, hydrodynamic radius, and density

The persistence length was obtained from the orientation correlation of segments along the polymer, expressed as a scalar product of two segment vectors separated by a contour length of L and predicted from the random coil statistics as an exponential decay governed by the persistence^85^. To estimate the persistence length of long simulated nucleosome array fibers containing *n* = 100 NCPs, the NCP pathway of the array was first converted into smoothly connected fixed length line segments (*d* = 15 *nm*). The persistence length was then estimated from the calculation of the autocorrelation decay along the trajectory of these line segments using the polymer.PersistenceLength.run script from MDAnalysis ^86^. The segment length (r) was defined by the distance between two distal ends of the line segment (*d* = 15 *nm*) and the resulted exponential decay fitting were averaged form 100 simulated array models. The hydrodynamic radius of the fiber was calculated from its persistence using the following equation based on worm-like chain model:

$$R_{g}^{2}=\frac{1}{3}PL\left[1-\frac{P}{L}\left(1-e^{-\frac{L}{P}}\right)\right]$$

where *R_g_* is the radius of gyration of the polymer, P is the persistence length of the simulated fiber, and L is the contour length of the fiber which is proportional to the number of line segments (*L* = *Nd*), which in turn is the number of threaded nucleosomes. The hydrodynamic radius *R_h_* is related to the radius of gyration *R_g_* by the equation: *R_h_*=0.662*R_g_*. The fiber density was calculated as: $n∕(\frac{4}{3}\pi R_{g}^{\;\;3})$.

## Extended Data

## References

[1] Ishihara S. Local states of chromatin compaction at transcription start sites control transcription levels. Nucleic acids research 49, 8007–8023, doi:10.1093/nar/gkab587 (2021). 34233004 PMC8373074

[2] Olins A. L. & Olins D. E. Spheroid chromatin units (v bodies). Science 183, 330–332, doi:10.1126/science.183.4122.330 (1974). 4128918

[3] Luger K., Mader A. W., Richmond R. K., Sargent D. F. & Richmond T. J. Crystal structure of the nucleosome core particle at 2.8 angstrom resolution. Nature 389, 251–260 (1997). 9305837 10.1038/38444

[4] Maeshima K., Imai R., Tamura S. & Nozaki T. Chromatin as dynamic 10-nm fibers. Chromosoma 123, 225–237 (2014). 24737122 10.1007/s00412-014-0460-2 PMC4031381

[5] Strickfaden H. Condensed Chromatin Behaves like a Solid on the Mesoscale In Vitro and in Living Cells. Cell 183, 1772–1784 e1713, doi:10.1016/j.cell.2020.11.027 (2020). 33326747

[6] Ohno M., Priest D. G. & Taniguchi Y. Nucleosome-level 3D organization of the genome. Biochem Soc Trans 46, 491–501, doi:10.1042/BST20170388 (2018). 29626147

[7] Robinson P. J., Fairall L., Huynh V. A. & Rhodes D. EM measurements define the dimensions of the "30-nm" chromatin fiber: evidence for a compact, interdigitated structure. Proceedings of the National Academy of Sciences of the United States of America 103, 6506–6511, doi:10.1073/pnas.0601212103 (2006). 16617109 PMC1436021

[8] Woodcock C. L., Frado L. L. & Rattner J. B. The higher-order structure of chromatin: evidence for a helical ribbon arrangement. The Journal of cell biology 99, 42–52, doi:10.1083/jcb.99.1.42 (1984). 6736132 PMC2275637

[9] Dorigo B. Nucleosome arrays reveal the two-start organization of the chromatin fiber. Science 306, 1571–1573, doi:10.1126/science.1103124 (2004). 15567867

[10] Garcia-Saez I. Structure of an H1-Bound 6-Nucleosome Array Reveals an Untwisted Two-Start Chromatin Fiber Conformation. Molecular cell 72, 902–915 e907, doi:10.1016/j.molcel.2018.09.027 (2018). 30392928

[11] Williams S. P. Chromatin fibers are left-handed double helices with diameter and mass per unit length that depend on linker length. Biophys J 49, 233–248, doi:10.1016/S0006-3495(86)83637-2 (1986). 3955173 PMC1329627

[12] Ou H. D. ChromEMT: Visualizing 3D chromatin structure and compaction in interphase and mitotic cells. Science 357, doi:10.1126/science.aag0025 (2017). PMC5646685 28751582

[13] Kireeva N., Lakonishok M., Kireev I., Hirano T. & Belmont A. S. Visualization of early chromosome condensation: a hierarchical folding, axial glue model of chromosome structure. The Journal of cell biology 166, 775–785, doi:10.1083/jcb.200406049 (2004). 15353545 PMC2172117

[14] Dehghani H., Dellaire G. & Bazett-Jones D. P. Organization of chromatin in the interphase mammalian cell. Micron 36, 95–108, doi:10.1016/j.micron.2004.10.003 (2005). 15629642

[15] Daban J. R. Stacked thin layers of metaphase chromatin explain the geometry of chromosome rearrangements and banding. Sci Rep 5, 14891, doi:10.1038/srep14891 (2015). 26446309 PMC4597206

[16] Daban J. R. The energy components of stacked chromatin layers explain the morphology, dimensions and mechanical properties of metaphase chromosomes. J R Soc Interface 11, 20131043, doi:10.1098/rsif.2013.1043 (2014). 24402918 PMC3899872

[17] Eltsov M., Maclellan K. M., Maeshima K., Frangakis A. S. & Dubochet J. Analysis of cryo-electron microscopy images does not support the existence of 30-nm chromatin fibers in mitotic chromosomes in situ. Proceedings of the National Academy of Sciences of the United States of America 105, 19732–19737, doi:10.1073/pnas.0810057105 (2008). 19064912 PMC2604964

[18] Maeshima K., Hihara S. & Eltsov M. Chromatin structure: does the 30-nm fibre exist in vivo? Curr Opin Cell Biol 22, 291–297, doi:10.1016/j.ceb.2010.03.001 (2010). 20346642

[19] Maeshima K., Ide S., Hibino K. & Sasai M. Liquid-like behavior of chromatin. Curr Opin Genet Dev 37, 36–45, doi:10.1016/j.gde.2015.11.006 (2016). 26826680

[20] Liu J. Fully Mechanically Controlled Automated Electron Microscopic Tomography. Sci Rep 6, 29231, doi:10.1038/srep29231 (2016). 27403922 PMC4941525

[21] Gibson B. A. Organization of Chromatin by Intrinsic and Regulated Phase Separation. Cell 179, 470–484 e421, doi:10.1016/j.cell.2019.08.037 (2019). 31543265 PMC6778041

[22] Strom A. R. Phase separation drives heterochromatin domain formation. Nature 547, 241–245, doi:10.1038/nature22989 (2017). 28636597 PMC6022742

[23] Hall M. A. High-resolution dynamic mapping of histone-DNA interactions in a nucleosome. Nature structural & molecular biology 16, 124–129, doi:10.1038/nsmb.1526 (2009). PMC2635915 19136959

[24] Song F. Cryo-EM study of the chromatin fiber reveals a double helix twisted by tetranucleosomal units. Science 344, 376–380, doi:10.1126/science.1251413 (2014). 24763583

[25] Bednar J. Structure and Dynamics of a 197 bp Nucleosome in Complex with Linker Histone H1. Molecular cell 66, 384–397 e388, doi:10.1016/j.molcel.2017.04.012 (2017). 28475873 PMC5508712

[26] Bilokapic S., Strauss M. & Halic M. Histone octamer rearranges to adapt to DNA unwrapping. Nat Struct Mol Biol 25, 101–108, doi:10.1038/s41594-017-0005-5 (2018). 29323273 PMC5800490

[27] Sanulli S. HP1 reshapes nucleosome core to promote phase separation of heterochromatin. Nature 575, 390–394, doi:10.1038/s41586-019-1669-2 (2019). 31618757 PMC7039410

[28] Zhang M. Molecular organization of the early stages of nucleosome phase separation visualized by cryo-electron tomography. Molecular cell 82, 3000–3014 e3009, doi:10.1016/j.molcel.2022.06.032 (2022). 35907400 PMC9493104

[29] Farr S. E., Woods E. J., Joseph J. A., Garaizar A. & Collepardo-Guevara R. Nucleosome plasticity is a critical element of chromatin liquid-liquid phase separation and multivalent nucleosome interactions. Nature communications 12, 2883, doi:10.1038/s41467-021-23090-3 (2021). PMC8129070 34001913

[30] Hihara S. Local nucleosome dynamics facilitate chromatin accessibility in living mammalian cells. Cell Rep 2, 1645–1656, doi:10.1016/j.celrep.2012.11.008 (2012). 23246002

[31] Cooper G. M., Hausman R. E. & Hausman R. E. The cell: a molecular approach. Vol. 4 (ASM press Washington, DC, 2007).

[32] Cai S. Cryo-ET reveals the macromolecular reorganization of S. pombe mitotic chromosomes in vivo. Proceedings of the National Academy of Sciences of the United States of America 115, 10977–10982, doi:10.1073/pnas.1720476115 (2018). 30297429 PMC6205422

[33] Maeshima K. Nucleosomal arrays self-assemble into supramolecular globular structures lacking 30-nm fibers. The EMBO journal 35, 1115–1132 (2016). 27072995 10.15252/embj.201592660 PMC4868957

[34] Marini M. The structure of DNA by direct imaging. Sci Adv 1, e1500734, doi:10.1126/sciadv.1500734 (2015). 26601243 PMC4643809

[35] Irobalieva R. N. Structural diversity of supercoiled DNA. Nat Commun 6, 8440, doi:10.1038/ncomms9440 (2015). 26455586 PMC4608029

[36] Beel A. J., Azubel M., Mattei P. J. & Kornberg R. D. Structure of mitotic chromosomes. Molecular cell 81, 4369–4376 e4363, doi:10.1016/j.molcel.2021.08.020 (2021). 34520722 PMC8571045

[37] Zhang L. & Ren G. IPET and FETR: experimental approach for studying molecular structure dynamics by cryo-electron tomography of a single-molecule structure. PloS one 7, e30249, doi:10.1371/journal.pone.0030249 (2012). 22291925 PMC3265479

[38] Buchholz T. O. Content-aware image restoration for electron microscopy. Methods Cell Biol 152, 277–289, doi:10.1016/bs.mcb.2019.05.001 (2019). 31326025

[39] Zhai X. LoTToR: An Algorithm for Missing-Wedge Correction of the Low-Tilt Tomographic 3D Reconstruction of a Single-Molecule Structure. Sci Rep 10, 10489, doi:10.1038/s41598-020-66793-1 (2020). 32591588 PMC7320192

[40] Phillips J. C. Scalable molecular dynamics on CPU and GPU architectures with NAMD. The Journal of chemical physics 153, 044130, doi:10.1063/5.0014475 (2020). 32752662 PMC7395834

[41] Polach K. J. & Widom J. Mechanism of Protein Access to Specific DNA-Sequences in Chromatin - a Dynamic Equilibrium-Model for Gene-Regulation. Journal of molecular biology 254, 130–149, doi:DOI 10.1006/jmbi.1995.0606 (1995). 7490738

[42] Winogradoff D. & Aksimentiev A. Molecular Mechanism of Spontaneous Nucleosome Unraveling. Journal of molecular biology 431, 323–335, doi:10.1016/j.jmb.2018.11.013 (2019). 30468737 PMC6331254

[43] Ngo T. T., Zhang Q., Zhou R., Yodh J. G. & Ha T. Asymmetric unwrapping of nucleosomes under tension directed by DNA local flexibility. Cell 160, 1135–1144, doi:10.1016/j.cell.2015.02.001 (2015). 25768909 PMC4409768

[44] Pedregosa F. Scikit-learn: Machine learning in Python. the Journal of machine Learning research 12, 2825–2830 (2011).

[45] Grigoryev S. A., Bednar J. & Woodcock C. L. MENT, a heterochromatin protein that mediates higher order chromatin folding, is a new serpin family member. The Journal of biological chemistry 274, 5626–5636, doi:10.1074/jbc.274.9.5626 (1999). 10026180

[46] Cai S., Bock D., Pilhofer M. & Gan L. The in situ structures of mono-, di-, and trinucleosomes in human heterochromatin. Mol Biol Cell 29, 2450–2457, doi:10.1091/mbc.E18-05-0331 (2018). 30091658 PMC6233054

[47] Ng C. T. & Gan L. Investigating eukaryotic cells with cryo-ET. Molecular biology of the cell 31, 87–100, doi:10.1091/mbc.E18-05-0329 (2020). 31935172 PMC6960407

[48] Schalch T., Duda S., Sargent D. F. & Richmond T. J. X-ray structure of a tetranucleosome and its implications for the chromatin fibre. Nature 436, 138–141, doi:10.1038/nature03686 (2005). 16001076

[49] Bhardwaj S. K. Dinucleosome specificity and allosteric switch of the ISW1a ATP-dependent chromatin remodeler in transcription regulation. Nature communications 11, 5913, doi:10.1038/s41467-020-19700-1 (2020). PMC7680125 33219211

[50] Dechassa M. L. SWI/SNF has intrinsic nucleosome disassembly activity that is dependent on adjacent nucleosomes. Molecular cell 38, 590–602, doi:10.1016/j.molcel.2010.02.040 (2010). 20513433 PMC3161732

[51] Gebala M., Johnson S. L., Narlikar G. J. & Herschlag D. Ion counting demonstrates a high electrostatic field generated by the nucleosome. Elife 8, doi:10.7554/eLife.44993 (2019). PMC6584128 31184587

[52] Manning G. S. Is a small number of charge neutralizations sufficient to bend nucleosome core DNA onto its superhelical ramp? Journal of the American Chemical Society 125, 15087–15092 (2003). 14653743 10.1021/ja030320t

[53] Yamada K. Structure and mechanism of the chromatin remodelling factor ISW1a. Nature 472, 448–453, doi:10.1038/nature09947 (2011). 21525927

[54] Ding X., Lin X. & Zhang B. Stability and folding pathways of tetra-nucleosome from six-dimensional free energy surface. Nature communications 12, 1091, doi:10.1038/s41467-021-21377-z (2021). PMC7889939 33597548

[55] Blank T. A. & Becker P. B. Electrostatic mechanism of nucleosome spacing. Journal of molecular biology 252, 305–313, doi:10.1006/jmbi.1995.0498 (1995). 7563052

[56] Turner A. L. Highly disordered histone H1− DNA model complexes and their condensates. Proceedings of the National Academy of Sciences 115, 11964–11969 (2018). 10.1073/pnas.1805943115 PMC6255207 30301810

[57] Li M. & Wang M. D. Unzipping single DNA molecules to study nucleosome structure and dynamics. Methods in enzymology 513, 29–58, doi:10.1016/B978-0-12-391938-0.00002-1 (2012). 22929764 PMC5515249

[58] Anderson J. D., Thastrom A. & Widom J. Spontaneous access of proteins to buried nucleosomal DNA target sites occurs via a mechanism that is distinct from nucleosome translocation. Mol Cell Biol 22, 7147–7157, doi:10.1128/MCB.22.20.7147-7157.2002 (2002). 12242292 PMC139820

[59] Anderson J. D. & Widom J. Sequence and position-dependence of the equilibrium accessibility of nucleosomal DNA target sites. Journal of molecular biology 296, 979–987, doi:10.1006/jmbi.2000.3531 (2000). 10686097

[60] Dombrowski M., Engeholm M., Dienemann C., Dodonova S. & Cramer P. Histone H1 binding to nucleosome arrays depends on linker DNA length and trajectory. Nature structural & molecular biology 29, 493–501, doi:10.1038/s41594-022-00768-w (2022). PMC9113941 35581345

[61] Scheffer M. P., Eltsov M. & Frangakis A. S. Evidence for short-range helical order in the 30-nm chromatin fibers of erythrocyte nuclei. Proceedings of the National Academy of Sciences of the United States of America 108, 16992–16997, doi:10.1073/pnas.1108268108 (2011). 21969536 PMC3193215

[62] Konig P., Braunfeld M. B., Sedat J. W. & Agard D. A. The three-dimensional structure of in vitro reconstituted Xenopus laevis chromosomes by EM tomography. Chromosoma 116, 349–372 (2007). 17333236 10.1007/s00412-007-0101-0

[63] McDowall A. W., Smith J. M. & Dubochet J. Cryo-electron microscopy of vitrified chromosomes in situ. The EMBO journal 5, 1395–1402 (1986). 3755397 10.1002/j.1460-2075.1986.tb04373.x PMC1166954

[64] Lai W. K. M. & Pugh B. F. Understanding nucleosome dynamics and their links to gene expression and DNA replication. Nature reviews. Molecular cell biology 18, 548–562, doi:10.1038/nrm.2017.47 (2017). 28537572 PMC5831138

[65] Liu G., Xing Y., Zhao H., Cai L. & Wang J. The implication of DNA bending energy for nucleosome positioning and sliding. Scientific reports 8, 1–12 (2018). 29891930 10.1038/s41598-018-27247-x PMC5995830

[66] Wittmeyer J., Saha A. & Cairns B. DNA translocation and nucleosome remodeling assays by the RSC chromatin remodeling complex. Methods Enzymol 377, 322–343, doi:10.1016/S0076-6879(03)77020-7 (2004). 14979035

[67] Rames M., Yu Y. & Ren G. Optimized negative staining: a high-throughput protocol for examining small and asymmetric protein structure by electron microscopy. Journal of visualized experiments : JoVE, e51087, doi:10.3791/51087 (2014). 25145703 PMC4710468

[68] Schorb M., Haberbosch I., Hagen W. J. H., Schwab Y. & Mastronarde D. N. Software tools for automated transmission electron microscopy. Nat Methods 16, 471–477, doi:10.1038/s41592-019-0396-9 (2019). 31086343 PMC7000238

[69] Zheng S. Q. MotionCor2: anisotropic correction of beam-induced motion for improved cryo-electron microscopy. Nat Methods 14, 331–332, doi:10.1038/nmeth.4193 (2017). 28250466 PMC5494038

[70] Mindell J. A. & Grigorieff N. Accurate determination of local defocus and specimen tilt in electron microscopy. Journal of structural biology 142, 334–347 (2003). 12781660 10.1016/s1047-8477(03)00069-8

[71] Frank J. SPIDER and WEB: processing and visualization of images in 3D electron microscopy and related fields. Journal of structural biology 116, 190–199 (1996). 8742743 10.1006/jsbi.1996.0030

[72] Zhang K. Gctf: Real-time CTF determination and correction. J Struct Biol 193, 1–12, doi:10.1016/j.jsb.2015.11.003 (2016). 26592709 PMC4711343

[73] Fernandez J. J., Li S. & Crowther R. A. CTF determination and correction in electron cryotomography. Ultramicroscopy 106, 587–596, doi:10.1016/j.ultramic.2006.02.004 (2006). 16616422

[74] Kremer J. R., Mastronarde D. N. & McIntosh J. R. Computer visualization of three-dimensional image data using IMOD. J Struct Biol 116, 71–76, doi:10.1006/jsbi.1996.0013 (1996). 8742726

[75] Ludtke S. J., Baldwin P. R. & Chiu W. EMAN: semiautomated software for high-resolution single-particle reconstructions. J Struct Biol 128, 82–97, doi:10.1006/jsbi.1999.4174 (1999). 10600563

[76] Yan R., Venkatakrishnan S. V., Liu J., Bouman C. A. & Jiang W. MBIR: A cryo-ET 3D reconstruction method that effectively minimizes missing wedge artifacts and restores missing information. J Struct Biol 206, 183–192, doi:10.1016/j.jsb.2019.03.002 (2019). 30872095 PMC6502674

[77] Pettersen E. F. UCSF Chimera--a visualization system for exploratory research and analysis. J Comput Chem 25, 1605–1612, doi:10.1002/jcc.20084 (2004). 15264254

[78] Hornus S., Levy B., Lariviere D. & Fourmentin E. Easy DNA modeling and more with GraphiteLifeExplorer. PloS one 8, e53609, doi:10.1371/journal.pone.0053609 (2013). 23308263 PMC3538550

[79] Lowary P. T. & Widom J. New DNA sequence rules for high affinity binding to histone octamer and sequence-directed nucleosome positioning. Journal of molecular biology 276, 19–42, doi:10.1006/jmbi.1997.1494 (1998). 9514715

[80] Rychkov G. N. Partially Assembled Nucleosome Structures at Atomic Detail. Biophys J 112, 460–472, doi:10.1016/j.bpj.2016.10.041 (2017). 28038734 PMC5300784

[81] Silverman B. W. Weak and Strong Uniform Consistency of the Kernel Estimate of a Density and its Derivatives. . The Annals of Statistics 6, , 177–184 (1978).

[82] Humphrey W., Dalke A. & Schulten K. VMD: visual molecular dynamics. J Mol Graph 14, 33–38, 27–38, doi:10.1016/0263-7855(96)00018-5 (1996). 8744570

[83] Levitt M. How many base-pairs per turn does DNA have in solution and in chromatin? Some theoretical calculations. Proceedings of the National Academy of Sciences of the United States of America 75, 640–644, doi:10.1073/pnas.75.2.640 (1978). 273227 PMC411311

[84] Bar-Joseph Z., Gifford D. K. & Jaakkola T. S. Fast optimal leaf ordering for hierarchical clustering. Bioinformatics 17 Suppl 1, S22–29, doi:10.1093/bioinformatics/17.suppl_1.s22 (2001). 11472989

[85] Cifra P. Differences and limits in estimates of persistence length for semi-flexible macromolecules. Polymer 45, 5995–6002, doi:10.1016/j.polymer.2004.06.034 (2004).

[86] Michaud-Agrawal N., Denning E. J., Woolf T. B. & Beckstein O. MDAnalysis: a toolkit for the analysis of molecular dynamics simulations. J Comput Chem 32, 2319–2327, doi:10.1002/jcc.21787 (2011). 21500218 PMC3144279

